# AMPK Activation through Mitochondrial Regulation Results in Increased Substrate Oxidation and Improved Metabolic Parameters in Models of Diabetes

**DOI:** 10.1371/journal.pone.0081870

**Published:** 2013-12-05

**Authors:** Yonchu Jenkins, Tian-Qiang Sun, Vadim Markovtsov, Marc Foretz, Wei Li, Henry Nguyen, Yingwu Li, Alison Pan, Gerald Uy, Lisa Gross, Kristen Baltgalvis, Stephanie L. Yung, Tarikere Gururaja, Taisei Kinoshita, Alexander Owyang, Ira J. Smith, Kelly McCaughey, Kathy White, Guillermo Godinez, Raniel Alcantara, Carmen Choy, Hong Ren, Rachel Basile, David J. Sweeny, Xiang Xu, Sarkiz D. Issakani, David C. Carroll, Dane A. Goff, Simon J. Shaw, Rajinder Singh, Laszlo G. Boros, Marc-André Laplante, Bruno Marcotte, Rita Kohen, Benoit Viollet, André Marette, Donald G. Payan, Todd M. Kinsella, Yasumichi Hitoshi

**Affiliations:** 1 Rigel Pharmaceuticals, Inc., South San Francisco, California, United States of America; 2 Inserm, U1016, Institut Cochin, Paris, France; 3 CNRS, UMR8104, Paris, France; 4 Université Paris Descartes, Sorbonne Paris cité, Paris, France; 5 SiDMAP, LLC, Los Angeles, California, United States of America; 6 Department of Pediatrics, Los Angeles Biomedical Research Institute (LABIOMED) at the Harbor-UCLA Medical Center, Torrance, California, United States of America; 7 Department of Medicine, Faculty of Medicine, Cardiology Axis of the Institut Universitaire de Cardiologie et de Pneumologie de Québec (Hôpital Laval), Québec, Québec, Canada; INSERM/UMR 1048, France

## Abstract

Modulation of mitochondrial function through inhibiting respiratory complex I activates a key sensor of cellular energy status, the 5'-AMP-activated protein kinase (AMPK). Activation of AMPK results in the mobilization of nutrient uptake and catabolism for mitochondrial ATP generation to restore energy homeostasis. How these nutrient pathways are affected in the presence of a potent modulator of mitochondrial function and the role of AMPK activation in these effects remain unclear. We have identified a molecule, named R419, that activates AMPK *in vitro* via complex I inhibition at much lower concentrations than metformin (IC_50_ 100 nM vs 27 mM, respectively). R419 potently increased myocyte glucose uptake that was dependent on AMPK activation, while its ability to suppress hepatic glucose production *in vitro* was not. In addition, R419 treatment of mouse primary hepatocytes increased fatty acid oxidation and inhibited lipogenesis in an AMPK-dependent fashion. We have performed an extensive metabolic characterization of its effects in the *db/db* mouse diabetes model. *In vivo* metabolite profiling of R419-treated *db/db* mice showed a clear upregulation of fatty acid oxidation and catabolism of branched chain amino acids. Additionally, analyses performed using both ^13^C-palmitate and ^13^C-glucose tracers revealed that R419 induces complete oxidation of both glucose and palmitate to CO_2_ in skeletal muscle, liver, and adipose tissue, confirming that the compound increases mitochondrial function *in vivo*. Taken together, our results show that R419 is a potent inhibitor of complex I and modulates mitochondrial function *in vitro* and in diabetic animals *in vivo*. R419 may serve as a valuable molecular tool for investigating the impact of modulating mitochondrial function on nutrient metabolism in multiple tissues and on glucose and lipid homeostasis in diabetic animal models.

## Introduction

Mitochondria are intracellular organelles devoted mainly to energy metabolism and perform a vital function in the production of ATP through oxidation of carbohydrates, fatty acids, and amino acids. Cumulative evidence has linked reduced mitochondrial function to the pathogenesis of diabetes and its complications [1,2]. However, over the past years, several studies have suggested that over activation of mitochondria is a potential risk for insulin resistance and that reduction of mitochondrial function may actually be protective under certain conditions [3-6], raising the possibility of mitochondrial modulation as a potential therapy for diabetes and its complications. Indeed, metformin, which is widely used for the treatment of type 2 diabetes, has been shown to directly inhibit complex I of the respiratory chain and reduce respiration with potencies in the high µM/low mM range [7,8]. In addition to metformin, several mitochondrial modulators have been reported to improve insulin sensitivity and metabolic complications [9,10]. Administration of such mitochondrial modulators results in activation of the 5'-AMP-activated protein kinase (AMPK), a master regulator of energy homeostasis, through increased AMP/ATP and ADP/ATP ratios. This results in initiation of both immediate and delayed responses that improve cellular capacity to restore ATP levels. Acutely, AMPK stimulates glucose transport and fatty acid oxidation in skeletal muscle [11,12]. Chronic adaptations to AMPK activation include up-regulation of proteins involved in substrate availability and oxidation capacity [13,14] and are integral to its role in enhancement of running endurance in normal mice [15,16]. Therefore, modulation of mitochondrial function is an attractive therapeutic option for diabetes and its complications. 

 Here, we have identified and characterized the activity of a novel small molecule inhibitor of complex I, R419, that potently activates AMPK in the low nM range. Our comprehensive analyses demonstrate that R419 treatment clearly altered mitochondrial function *in vivo*, resulting in increased branched chain amino acid catabolism and increased oxidation of both glucose and palmitate in three major metabolic organs, liver, skeletal muscle, and adipose tissue. These mitochondrial effects observed with R419 treatment reveal how small molecule modulation of mitochondrial function can lead to significant improvements in metabolic parameters in diabetic mouse models. 

## Materials and Methods

### Materials

Antibodies against phospho-acetyl-CoA-carboxylase (ACC) S79, total ACC, phospho-AMPK T172, total AMPK α1/2, phospho-UNC-51-like kinase (ULK) 1 S555, β-actin and glyceraldehyde-3-phosphate dehydrogenase (GAPDH) were from Cell Signaling Technologies. A-769662 was from Tocris Bioscience. AICAR and metformin were from Toronto Research Chemicals Inc. Rotenone, carbonylcyanide-p-trifluoromethoxyphenylhydrazone (FCCP), antimycin, and oligomycin were purchased from Seahorse Biosciences. All other reagents were purchased from Sigma-Aldrich unless indicated otherwise. NAD^+^/NADH was measured using a commercially available kit (Abcam Ltd.) according to manufacturer’s instructions.

### Cell culture

HepG2 cells (ATCC) were maintained in minimum essential Eagle medium supplemented with 10% FCS (Sigma-Aldrich). C2C12 myoblasts (ATCC) were maintained in Dulbecco’s modified Eagle medium (DMEM) supplemented with 10% FCS and differentiated by incubating confluent monolayers in DMEM containing 2% horse serum (Hyclone). XA15A1 human preadipocytes (Lonza) were maintained in complete SKGM-2 medium (Lonza) and differentiated by incubating confluent monolayers in DMEM containing PGM-2 supplements (Lonza). Primary muscle cells from wild type mice and mice lacking both AMPKα1 and AMPKα2 catalytic subunits (AMPK α1/α2 KO mice) were isolated and maintained as described previously [17]. Primary hepatocytes from both wild type mice and mice lacking both AMPKα1 and AMPKα2 catalytic subunits [18] were isolated from fed adult mice by a modified version of the collagenase method [19] and maintained as previously described [20]. 

### AMPK cell-based activation assay

HepG2 and C2C12 assays were performed as described previously [21] with the following adaptations. Non-starved cells plated 24 hours prior to treatment were dosed with compound for 2 hours at 37°C. After fixation with 4% formaldehyde in PBS (20 minutes), cells were incubated in 5% BSA in PBS containing 0.1% TritonX-100 (BSA-PBST) for one hour followed by incubation overnight at 4°C with anti-phospho-ACC (1:500) diluted in BSA-PBS. Cells were washed multiple times with PBST following removal of the primary antibody and then incubated with goat anti-rabbit-HRP diluted 1:9000 in PBS-BSA. The EC_50_ determination for both assays was executed using MatLab software version 6.5 (MathWorks, Inc.). Mean ± SD of EC_50_ values acquired from multiple experiments and representative EC_50_ curves are presented. 

### HPLC determination of adenine nucleotide ratios

HepG2 cells plated in 10 cm dishes were treated with compounds for 2 hours at 37°C. Cells were lysed in 10% trichloroacetic acid following a PBS wash and then transferred to microfuge tubes. Cleared lysates were extracted using water-saturated diethyl ether, followed by neutralization with 2.5 M KOH. Ten µl of each lysate was analyzed by reverse phase HPLC using a gradient consisting of 20 mM potassium dihydrogen phosphate (KH_2_PO_4_)/10 mM tetrabutylammonium hydroxide (C_4_H_9_)_4_NOH, pH 8.5 and 100 mM KH_2_PO_4_/30% methanol/10 mM (C_4_H_9_)_4_NOH, pH 6.0 (Supelcosil LC-18-T, 15 cm x 4.6 mm ID column, 0.7 mls/min flow rate, absorbance monitored at 254 nM). Nucleotide ratios were calculated using peak areas determined by Agilent software. Peaks were identified as AMP, ADP, and ATP according to comparison of retention times with standards as well as additional experimental runs spiked with internal standards.

### Respiration assays

All measurements of oxygen consumption rates were performed in 24 well plates using an XF24 Extracellular Flux Analyzer (Seahorse Bioscience). For experiments utilizing mouse liver mitochondria, 24 well plates containing 5 µg of freshly isolated mitochondria in 50 µL of MAS buffer (70 mM sucrose, 220 mM mannitol, 10 mM KH_2_PO_4_, 5 mM MgCl_2_, 2 mM HEPES, 1 mM EGTA and 0.2% fatty acid-free BSA, pH 7.2) per well were centrifuged at 2000 rpm (Beckman Allegra6R) for 20 minutes at 4°C prior to initiation of experiments. Mitochondria were isolated as previously described [22]. Briefly, livers were quickly excised from euthanized mice, transferred to a glass beaker placed on ice, rinsed with ice cold buffer containing 250 mM sucrose, 5 mM Tris, pH 7.4 and 2 mM EGTA (STE buffer) and then minced in 25 mls of STE buffer. The liver slurry was washed with STE buffer until clear, homogenized using 5-6 strokes of a dounce homogenizer and centrifuged 3 min at 1000 rpm. The supernatant was transferred to a fresh tube and centrifuged at 4°C for 10 minutes at 11600 rpm. The pellet was resuspended in STE buffer, centrifuged, and then resuspended to a final concentration of 50 mg/ml.

### Glucose uptake assays

Primary myotubes from wild type and AMPK α1/α2 KO mice were serum-starved for 2 hours prior to assaying glucose uptake and were exposed to 500 nM R419 or 2 mM metformin for the times indicated in the figure. Cells were washed three times with HEPES-buffered saline (HBS: 140 mM NaCl, 20 mM HEPES, 5 mM KCl, 2.5 mM MgSO_4_, 1 mM CaCl_2_, pH 7.4). Glucose uptake was assayed by incubation of 2-deoxy-D-[^3^H]glucose (1 µCi/ml, 26.2 Ci/mmol) for 10 min as described previously [23,24]. Nonspecific binding was determined by quantitating cell-associated radioactivity in the presence of 10 µM cytochalasin B. Radioactive medium was aspirated prior to washing cells three times with ice-cold saline. Cells were subsequently lysed in 50 mM NaOH, and radioactivity was quantitated using a Beckman LS 6000IC scintillation counter. Protein concentration in cell lysates was determined using the Bradford method [25]. The radioactivity was normalized to protein concentration in the cell lysate and the nonspecific binding was subtracted. The data at each time point is normalized to the corresponding vehicle control.

### Hepatocyte glucose production

After incubation in M199 medium containing 100 nM dexamethasone (dex) for 16 hours, hepatocytes were washed once with PBS and glucose production was determined after 8 hour-incubation in glucose-free DMEM containing lactate and pyruvate (10:1 mM) with/without 100 μM *N6,O2'*-dibutyryladenosine 3'5'-cyclic monophosphate (Bt2-cAMP) (Sigma-Aldrich) in the presence or absence of various doses of R419 or metformin. At the end of the incubation, 1.5 ml of medium was collected and the amount of glucose released into the medium was determined by evaluating the production of NADPH from NADP in the presence of hexokinase and glucose-6-phosphate dehydrogenase (Roche Applied Science) and normalized to the total protein content per well [20].

### Fatty acid oxidation assays

Primary mouse hepatocytes were plated with 5x10^4^ cells/well in 48-well plates and incubated overnight in M199 medium containing penicillin/streptomycin (Life technologies) and 100 nM dexamethasone prior to palmitate oxidation measurement [26]. Various concentrations of R419 in DMSO were added for 3 hours. Each well was pulse-labeled with 0.19 µCi [1-^14^C]palmitate (56.0 mCi/mmol) (Perkin Elmer) and 50 µM palmitic acid complexed to 0.56 % bovine serum albumin added directly in the media for an additional 90 min. Exogenous palmitate oxidation was monitored by measuring production of ^14^C-labeled acid-soluble metabolites (ASM), a measure of tricarboxylic acid cycle intermediates and acetyl esters. Reactions were terminated by aspiration of the media and addition of 400 μl of HClO_4_ at 5% for 45 min at room temperature. The ASM was assayed in supernatants of the acid precipitate. Radioactivity of ASM was determined by liquid scintillation counting by use of 4.5 ml of liquid scintillation cocktail (Optiphase “Hisafe” 3, Perkin Elmer) in scintillation vials. For protein determination, identical incubations were conducted on parallel plates with the same number of cells.

### Lipid synthesis assays

Primary mouse hepatocytes on 6-well plates (4x10^5^ cells/well) were maintained in M199 medium containing penicillin/streptomycin and 100 nM dexamethasone for 16 hours prior to the measurement of fatty acids synthesis. Hepatocytes were incubated for 3 hours with 0.6 µCi/ml [1-^14^C]acetate (55.3 mCi/mmol) (Perkin Elmer) in M199 medium with various doses of R419 compound or 10 µM TOFA. Controls consisted of cells incubated with DMSO alone. The cells were then scraped in 1ml of PBS and transferred to tubes containing 1 ml of 40% KOH. An equal volume of methanol was added and the tubes were heated at 80°C for 1 hour. The non-saponifiable fraction was removed by extraction into petroleum ether. The aqueous phase was then acidified with concentrated HCl. Fatty acids were extracted into petroleum ether; the extract was washed with 5% acetic acid and taken to dryness by steaming. The samples were counted using a liquid scintillation counter. Data were normalized to protein content and expressed as a percentage of control.

### Ethics statement

All animal studies were performed in accordance with Rigel IACUC guidelines (Institutional Animal Care and Use Committee of Rigel Pharmaceuticals, Inc.) and the institutional review board specifically approved this study (protocol number Rigel-2-2012).

### Animal care and treatment

Eight-week-old male *db/db* mice were obtained from Jackson Laboratories and acclimated for at least four days prior to study initiation. R419 was prepared in 0.5% hydroxypropyl methylcellulose/0.1% Tween80 (vehicle) for oral once daily dosing (PO QD). Mice were weighed and randomly assigned to dose groups so that the mean weight of every cage (34~35g average weight and 4 mice/cage) was roughly the same. Dosing was performed PO QD for all treatments in the morning for the time duration indicated in the figure legends. For blood and tissue collection, animals were sacrificed by carbon dioxide asphyxiation followed by cardiac venipuncture. After harvesting, tissues were immediately frozen in liquid nitrogen and stored at -80°C.

### Metabolomics study

Male *db/db* mice (8 weeks) were orally gavaged once daily with either vehicle or 10 mg/kg R419. Liver, muscle, adipose tissue, and plasma from treated mice (n = 6/treatment group) were collected 30 minutes after dosing both on day 3 of treatment. Frozen tissue and plasma samples were sent to Metabolon for unbiased metabolite analysis (Durham, NC) [27-29]. Biochemical data were analyzed using Welch’s two-sample t-tests.

### ^13^C-tracer analyses.

Male db/db mice (8 weeks) were orally gavaged once daily with vehicle, 5 mg/kg R419, or 10 mg/kg R419. Thirty minutes after dosing at day 8, 0.5 mg/kg of the metabolic tracer was administered, either via intraperitoneal injection for [U-^13^C]-glucose or oral gavage for [U-^13^C]-palmitate, followed by collection of liver, muscle, adipose tissue, and plasma at the timepoints indicated in the figure legends. Frozen samples were sent to SiDMAP, LLC (Los Angeles, CA) for isotope tracer analysis [30,31]. [U-^13^C]-D-glucose and [U-^13^C]-palmitate were from Sigma-Aldrich. Sample preparation, analysis, and informatics were performed on blinded samples.

### Statistical methods

All statistical analyses were performed using GraphPad Prism version 6.0. An unpaired 2-tailed Student’s t-test was used to assess statistical significance where one group was compared to control. One-way or two-way ANOVA analyses followed by ad-hoc tests were performed to compare multiple groups. Statistical methods are described in each figure legend. Statistical significance is defined as p < 0.05 or less and indicated using asterisks in the figures.

## Results

**Figure 1:**
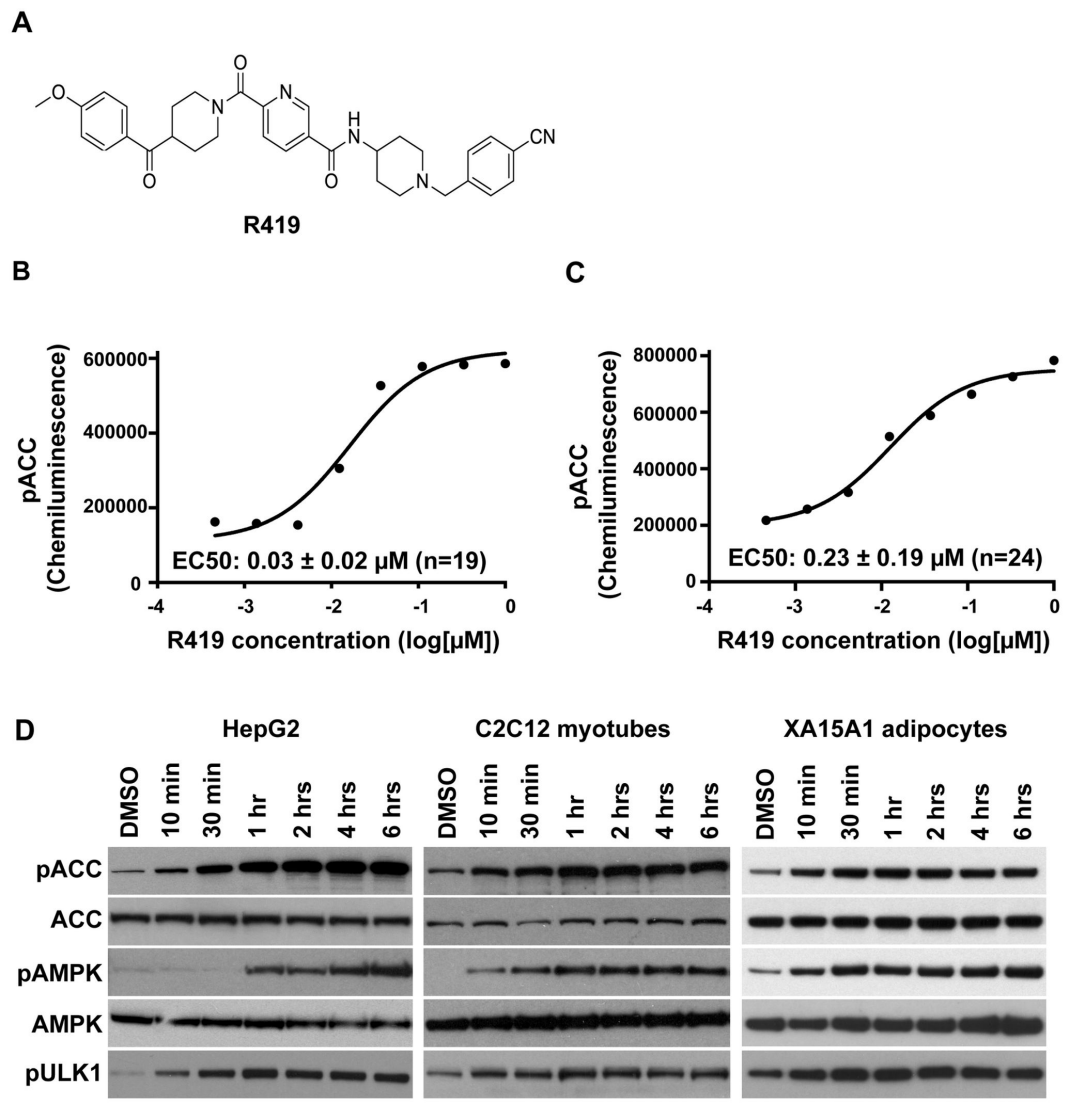
R419 structure and AMPK activation in HepG2 cells, C2C12 myotubes, and XA15A1 adipocytes. *A*: Chemical structure of R419. *B* and *C*: Dose response relationship between R419 and AMPK activation in HepG2 cells and C2C12 myotubes. Mean ± SD of EC_50_ values acquired from multiple experiments (the number of experiments is indicated in parentheses) and representative EC_50_ curves are presented. *D*: AMPK activation by R419 in HepG2 cells, C2C12 myotubes, and XA15A1 adipocytes. The cells were incubated with 0.2 µM R419 at 37°C for 2 hours. Lysates were blotted using the indicated antibodies.

R419 activates AMPK in liver, muscle, and adipose cells. The compound R419 (N-(1-(4-cyanobenzyl)piperidin-4-yl)-6-(4-(4-methoxybenzoyl)piperidine-1-carbonyl)nicotinamide, molecular mass of 565.67, international application publication no. WO 2012/016217, February 2, 2012) (Figure 1A) is a representative example of a potent series of small molecules identified through structure activity relationship studies as AMPK activators using upregulation of substrate ACC S79 phosphorylation in HepG2 liver cells as a readout. Dose response curves in HepG2 cells and C2C12 myotubes yielded EC_50_s of 0.03±0.02 µM and 0.23±0.19 µM, respectively (Figure 1B and 1C). Further analysis of P-AMPK kinetics as well as phosphorylation of AMPK substrates (ACC and ULK1) in both cell types as well as XA15A1 adipocytes revealed an increase in substrate phosphorylation within ten minutes that plateaus between 0.5 to 2 hours (Figure 1D), demonstrating that AMPK activation by R419 is rapid and occurs in cell lines representing the three major metabolic organs.

### R419 activates AMPK through inhibition of mitochondrial complex I

**Figure 2:**
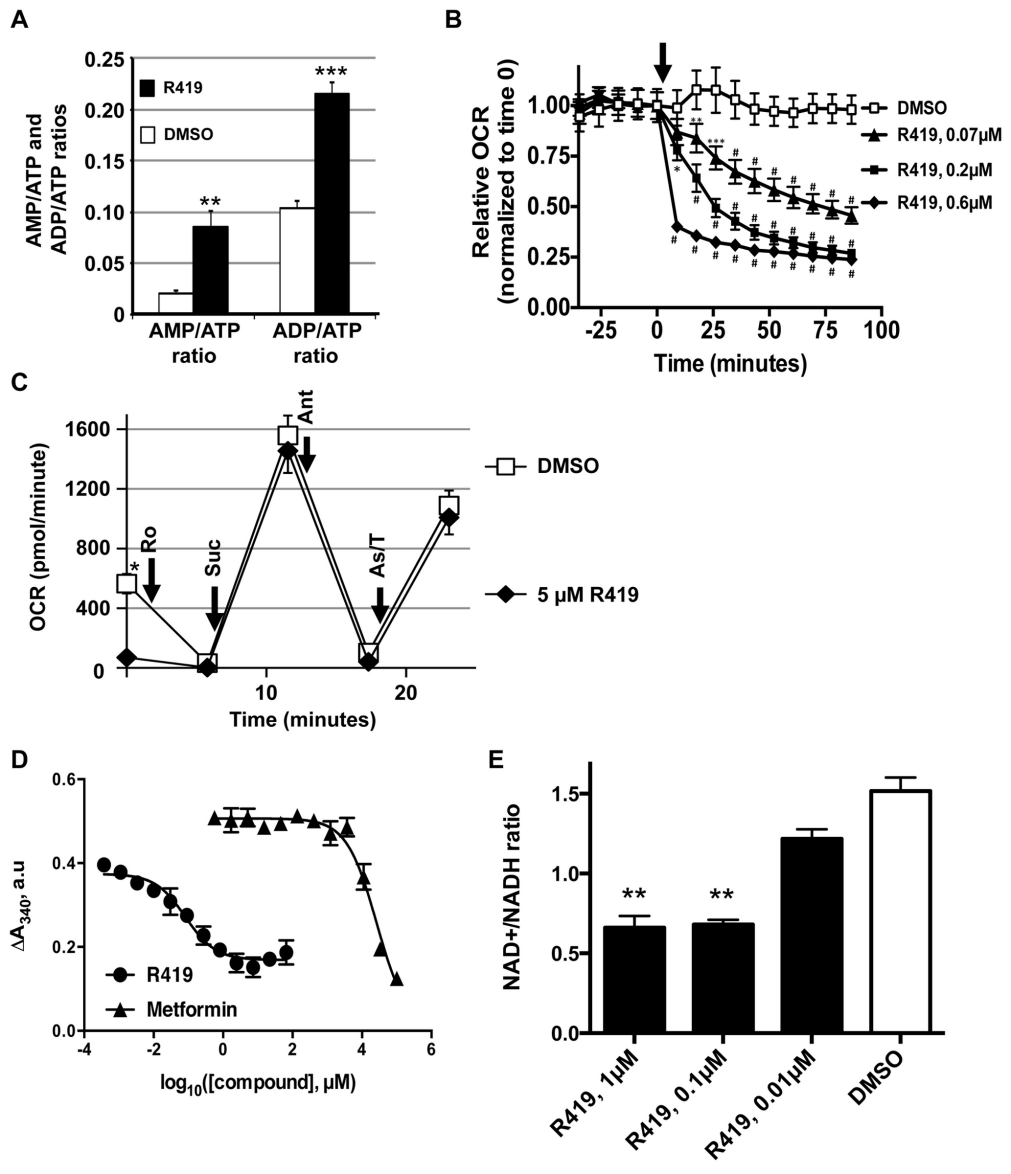
Reduction of mitochondrial respiration by R419 through complex I inhibition. *A*: Increase in AMP/ATP and ADP/ATP ratios in HepG2 cells. The cells were treated with 0.05% DMSO (open bar) or 200 nM R419 (solid bar) for 6 hours. Nucleotide levels were measured by HPLC. The data are presented as mean (bar) ± SEM (line) of triplicate cultures. Unpaired two-tailed t-tests were performed between DMSO control and R419-treated group. Asterisks ** and *** represent p < 0.01 and p < 0.001. *B*: Dose-dependent reduction of oxygen consumption rate (OCR) in HepG2 cells. OCR in the presence of DMSO or R419 was measured using an XF24 Seahorse instrument. Time point of R419 or DMSO injection is indicated by arrow. The data are presented as mean (symbol) ± SEM (line) of triplicate cultures. Repeated measures two-way ANOVA followed by the Dunnett ad-hoc test was performed and the multiple comparisons were done against the corresponding DMSO control at each timepoint. Asterisks *, **, *** and # represent p < 0.05, p<0.01, p<0.001 and p < 0.0001. *C*: Intact succinate-driven respiration in the presence of R419 in purified mouse liver mitochondria. The assay was conducted using an XF24 Seahorse instrument according to manufacturer’s protocols. 5 µg of purified mitochondria per well were used. Rotenone (Ro), succinate (Suc), antimycin A (Ant) and ascorbate/N,N,N',N'-tetramethyl-p-phenylene diamine (As/T) mixture were introduced at timepoints indicated by arrows. DMSO (n=6) or R419 (n=4) was introduced immediately prior to the assay. The data are presented as mean (symbol) ± SEM (line). Repeated measures two-way ANOVA followed by the Sidak ad-hoc test was performed and the multiple comparison test was done against the corresponding DMSO control at each timepoint. Except for time 0, significant differences in OCR between the two groups were not observed. Asterisk * represents p < 0.01. *D*: Inhibition of complex I-mediated NADH oxidation by R419 in purified mouse liver mitochondria. R419 or metformin was added to a mitochondrial lysate preparation (330 µg/ml) containing 2 mM NADH and incubated for 20 minutes. NADH to NAD^+^ conversion was measured by monitoring the absorbance at 340 nm. Difference between the initial absorbance and the absorbance after 20-minute incubation was presented as ΔA340 a.u. (absorbance units). The data are presented as mean (symbol) ± range between two measures (line) of duplicate cultures. Statistical analyses were not performed for this Data Set. *E*: Reduction of NAD^+^/NADH ratio by R419. HepG2 cells were treated with 0.05% DMSO or R419 for 2 hours. NAD^+^ and NADH levels in cell lysates were measured using a commercially available kit. The data are presented as mean (bar) ± range between two measures (line) of duplicate cultures. Ordinary one-way ANOVA with the Dunnett ad-hoc test was performed. The multiple comparison test was done against the DMSO control. Asterisk ** represents p < 0.01.

Measurements of adenine nucleotide ratios in HepG2 cells treated with R419 revealed clear increases in both the AMP/ATP and the ADP/ATP ratios (Figure 2A), indicating that R419 affects cellular energy status. To evaluate potential effects of R419 on cellular ATP production, we measured oxygen utilization in HepG2 cells treated with compound (Figure 2B). Indeed, increasing R419 concentrations led to marked decreases in the oxygen consumption rate (OCR) compared to the DMSO control, with a dose-dependence similar to that observed for ACC S79 phosphorylation in the same cell line (Figure 1B). 

To elucidate the molecular basis of R419’s effect on ATP production, we measured the OCR of fully uncoupled mouse liver mitochondria in the presence of either DMSO or 5 µM R419 (Figure 2C). Similar to its effects on HepG2 cells, R419 inhibited the OCR in isolated mitochondria. However, the OCR was completely rescued by adding succinate to fuel respiration through complex II, thus restoring the electron flow to the level of DMSO control (Figure 2C). With succinate-driven respiration, identical behavior was observed for both treatments following sequential addition of antimycin A (complex III inhibitor) and ascorbate/N1,N1,N1,N1-tetramethyl-1,4-phenylene diamine (complex IV electron donor), showing that R419 is not an inhibitor of complexes II, III and IV and suggesting that the molecular target of R419 lies upstream of complex II, possibly within the tricarboxylic acid cycle (TCA) or complex I. We assessed R419 activity *in vitro* against individual TCA enzymes as well as complexes in mouse liver mitochondrial lysates. While R419 displayed no effects on the TCA cycle (Table S1), the complex I-mediated oxidation of NADH to NAD^+^ in the mitochondrial lysate was inhibited (IC_50_ of 82 nM) (Figure 2D). Metformin was a significantly weaker inhibitor of complex I activity (IC_50_ of 27 mM). In line with the observed biochemical inhibition of complex I, the NAD^+^/NADH ratio in lysates from R419-treated HepG2 cells was also reduced (Figure 2E). 

### R419 displays both AMPK-dependent and independent effects on glucose and fat metabolism *in vitro*

**Figure 3:**
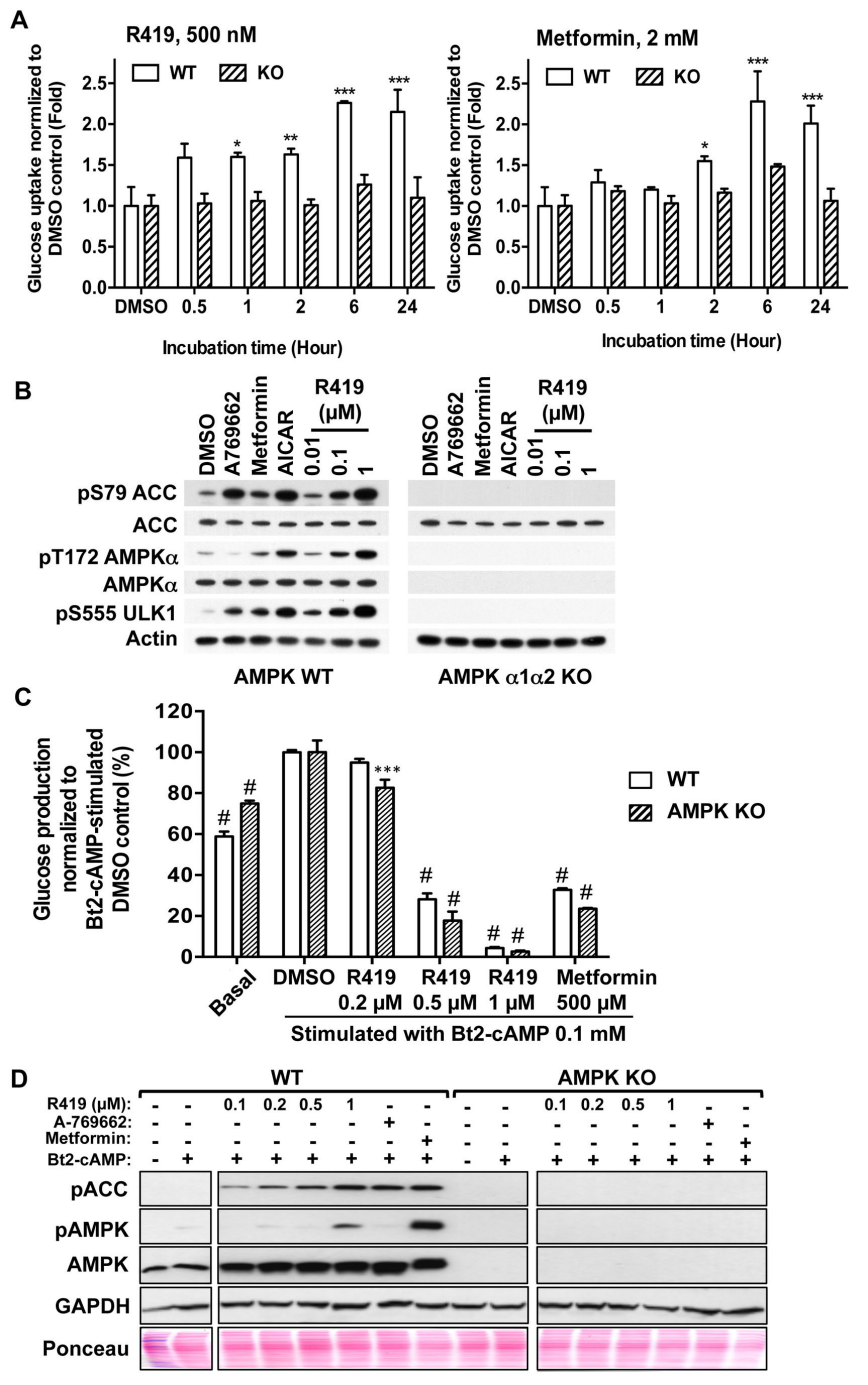
AMPK-dependent glucose uptake and AMPK-independent gluconeogenesis inhibition by R419. *A*: AMPK-dependent glucose uptake by R419 in primary mouse muscle cells. The cells were exposed to R419 or metformin for indicated times. Glucose uptake was assayed by incorporation of 2-deoxy-D-[^3^H]glucose (1%Ci/ml, 26.2 Ci/mmol) into the cell lysate in 10 min. The data are presented as mean (bar) ± SEM (line) of 2~8 individual experiments performed in triplicate (line) (n=8 (DMSO), n=3 (0.5 hour), n=4 (1 hour), n=7 (2 hours), n=2 (6 hours) and n=3 (24 hours)). Ordinary two-way ANOVA followed by the Dunnett ad-hoc test was performed and the multiple comparison test within the same genotype was done against each DMSO control. Asterisks *, ** and *** represent p<0.05, p<0.01 and p < 0.001, respectively. *B*: AMPK-dependent ACC and ULK1 phosphorylation by R419. Primary muscle cells from AMPK wild type mice and AMPK α1/α2 KO mice were treated with A-769662 (300 µM), metformin (5 mM), AICAR (2 mM), and R419 (0.01, 0.1 and 1 µM) for one hour. Lysates were blotted using the indicated antibodies. *C*: AMPK-independent suppression of glucose production by R419. Primary hepatocytes from WT mice and AMPK α1/α2 KO mice were stimulated with Bt2-cAMP in the presence or absence of R419 or metformin for eight hours. The amount of glucose released into the media was normalized to protein content. Data are normalized to DMSO control and presented as mean (bar) ± SEM (line) of triplicate cultures. Ordinary two-way ANOVA followed by the Dunnett ad-hoc test was performed and the multiple comparisons within the same genotype was done against each DMSO control. Significance against each DMSO control is indicated on top of the bars. Asterisks *** and # represent p<0.001 and p < 0.0001, respectively. Genotype (WT vs KO) is not a significant source of variation by ordinary two-way ANOVA. D: AMPK-dependent ACC and AMPK phosphorylation in hepatocytes by R419. Primary hepatocytes from wild type mice (WT) and AMPK α1/α2 KO mice (AMPK KO) were stimulated with 100 µM Bt2-cAMP in the presence or absence of R419 (0.1, 0.2, 0.5 and 1 µM), A-769662 (30 µM) or metformin (0.5 mM) for eight hours. Lysates were blotted using the indicated antibodies. Quantities of transferred protein on the membrane were examined with Ponceau S staining solution (Ponceau). The images of western blots and the Ponceau S-stained membrane are trimmed and different parts of the same blots are grouped.

We utilized primary cells from WT and AMPK α1/α2 KO mice to determine activity as well as AMPK dependence of R419 in several different assays of glucose and fat metabolism. Figure 3A compares the magnitude and time-course of glucose uptake by R419 with metformin in primary muscle cultures. Glucose uptake mediated by R419 occurs more rapidly than with metformin, with a substantial increase in glucose uptake occurring within 30 minutes for R419. In addition, the R419 activity appeared biphasic, with a further increase apparent at 6 hours of treatment that may reflect transcriptional changes induced by the compound. In support of this, GLUT4 promoter activity was upregulated in C2C12 myocytes by R419 treatment as shown in Figure S1. Glucose uptake was blunted or significantly reduced at all time points in the α1/α2 KO cells, indicating that glucose uptake induced by R419 and metformin is mostly AMPK dependent. 

These same cells were used to examine the AMPK dependence of substrate phosphorylation mediated by R419 as well as other reported AMPK activators A-769662, metformin, and AICAR (Figure 3B). The dose-dependent increases in P-AMPK, P-ULK1, and P-ACC observed for R419 in WT cells were completely abolished in the AMPK α1/α2 KO cells. While metformin and AICAR showed responses similar to R419, treatment with the direct AMPK activator A-769662 had no effect on AMPK T172 phosphorylation although AMPK kinase activity was clearly stimulated since substrate phosphorylation was elevated. The increase in phosphorylation of both the kinase and its substrates is consistent with indirect stimulation of activity via AMP generation by R419, which protects AMPK T172 from dephosphorylation. In the AMPK α1/α2 KO cells, substrate phosphorylation was abrogated for all the AMPK activators tested, confirming that induction of ACC and ULK1 phosphorylation depends completely on AMPK activation in muscle cells.

We also examined the activity of R419 on gluconeogenesis in mouse primary hepatocyte cultures. Suppression of hepatic glucose production is thought to be the primary mechanism contributing to metformin’s ability to maintain glucose homeostasis. Reduction of gluconeogenesis by metformin was recently attributed to a decrease in hepatic energy charge, rather than AMPK activation [20]. In WT hepatocytes, both R419 and metformin inhibited cumulative glucose production stimulated by the cAMP analog dibutyryl-cAMP (Bt2-cAMP) (Figure 3C). The inhibitory effect on cAMP-stimulated glucose production was dose-dependent with an IC_50_ between 200 and 500 nM R419. As expected based both upon results reported for metformin and also on our R419 mechanistic studies, the ability of R419 to suppress cAMP–stimulated glucose production was maintained in primary AMPK α1/α2 KO hepatocytes. Western blotting analysis of AMPK WT and α1/α2 KO hepatocytes treated with R419 showed that P-AMPK and substrate P-ACC are completely dependent upon the presence of AMPK (Figure 3D) and confirms that gluconeogenesis suppression by R419 does not require AMPK.

**Figure 4:**
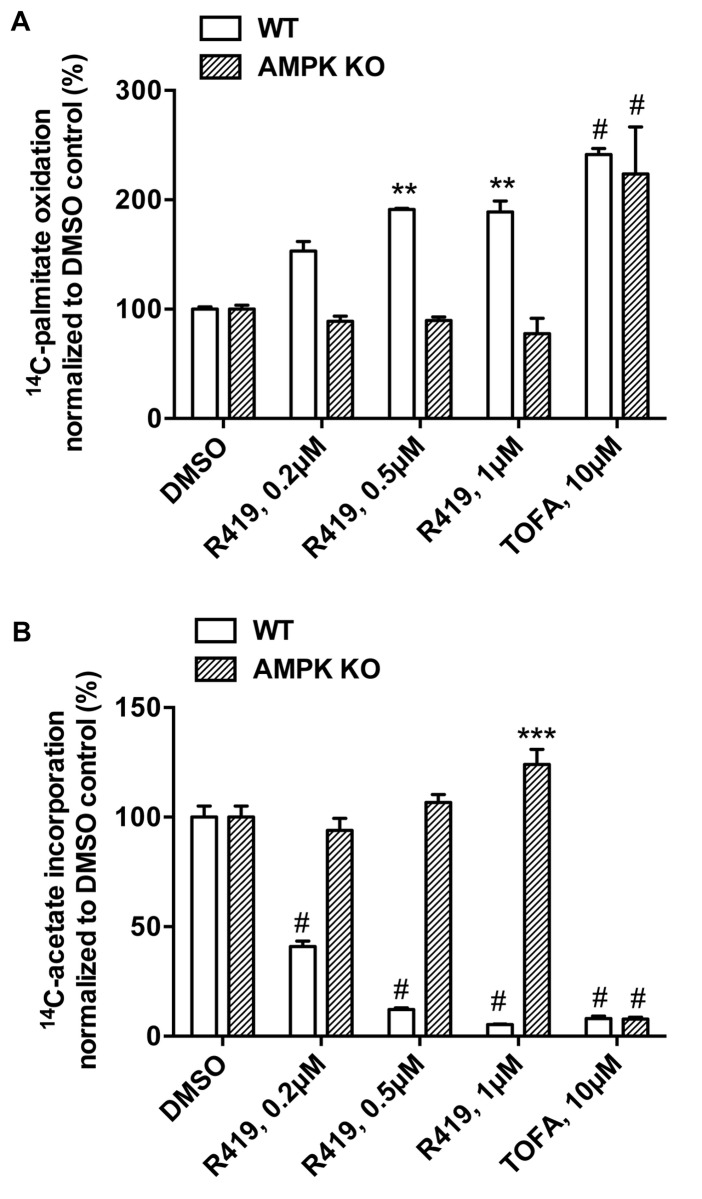
AMPK dependence of R419 mediated fatty acid oxidation and inhibition of fatty acid synthesis. *A*: Primary hepatocytes from wild type mice (WT: open bar) and AMPK α1/α2 KO mice (AMPK KO: hatched bar) were cultured in the presence or absence of R419 (0.2, 0.5 and 1 µM), or TOFA (10 µM) for three hours. Exogenous palmitate oxidation was monitored by the production of ^14^C-labeled acid-soluble metabolites as described in materials and methods. The data are presented as mean (bar) ± SEM (line) of triplicate cultures. Ordinary two-way ANOVA followed by the Dunnett ad-hoc test was performed for statistical analyses and the multiple comparisons within the same genotype were performed against each DMSO control. Significance against each DMSO control is indicated on top of the bars. Asterisks ** and # represent p < 0.01 and p < 0.0001. *B*: Primary hepatocytes from wild type mice (WT: open bar) and AMPK α1/α2 KO mice (AMPK KO: hatched bar) were cultured in the presence or absence of R419 (0.2, 0.5 and 1 µM), or TOFA (10 µM) for three hours. Acetate incorporation was monitored by the production of ^14^C-labeled lipids as described in materials and methods. The data are presented as mean (bar) ± SEM (line) of triplicate cultures. Ordinary two-way ANOVA followed by the Dunnett ad-hoc test was performed for statistical analyses and the multiple comparisons within the same genotype were performed against each DMSO control. Significance against each DMSO control is indicated on top of the bars. Asterisks *** and # represent p < 0.001, and p < 0.0001.

Since ACC is a key regulatory enzyme for fatty acid metabolism, we examined R419 effects on both fatty acid oxidation and synthesis in primary mouse hepatocytes. When wild type cells were treated with R419, palmitate oxidation into acid-soluble metabolites was increased in a dose-dependent manner with the maximal response reached at 500 nM concentration (Figure 4A). When [1-^14^C]acetate was added to primary hepatocyte cultures to probe fatty acid synthesis, a significant dose-dependent inhibition of acetate incorporation was observed in WT cells treated with R419 (Figure 4B). R419-mediated effects on both β-oxidation and lipogenesis are AMPK-dependent as complete abrogation of R419 activity was observed in the α1/α2 KO hepatocytes, which is consistent with the lack of ACC phosphorylation in the KO cells by R419. Treatment of hepatocytes with 5-tetradecyloxy-2-furoic acid (TOFA), a competitive inhibitor of ACC, also displayed similar effects on fatty acid oxidation and synthesis but unlike R419, the TOFA-mediated activities were apparent in both WT and α1/α2 KO hepatocytes.

### R419 treatment affects mitochondrial function *in vivo*.

We performed metabolite profiling of tissues from *db/db* mice orally dosed with either vehicle or 10 mg/kg R419 to characterize in detail the effects of R419 on nutrient pathways in a commonly used mouse model of type 2 diabetes. The R419 dose was selected based upon the robust AMPK activation observed in both liver and muscle lysates from normal C57BL/6J mice given a single oral administration of 10 mg/kg R419 (Figure S2). Since AMPK activation results in both acute and chronic changes to metabolic pathways, we chose to analyze samples from non-fasted mice treated for three days with R419. Samples were collected thirty minutes after compound dosing when R419 plasma levels as well as tissue AMPK activation were maximal (Figure S3).

**Figure 5:**
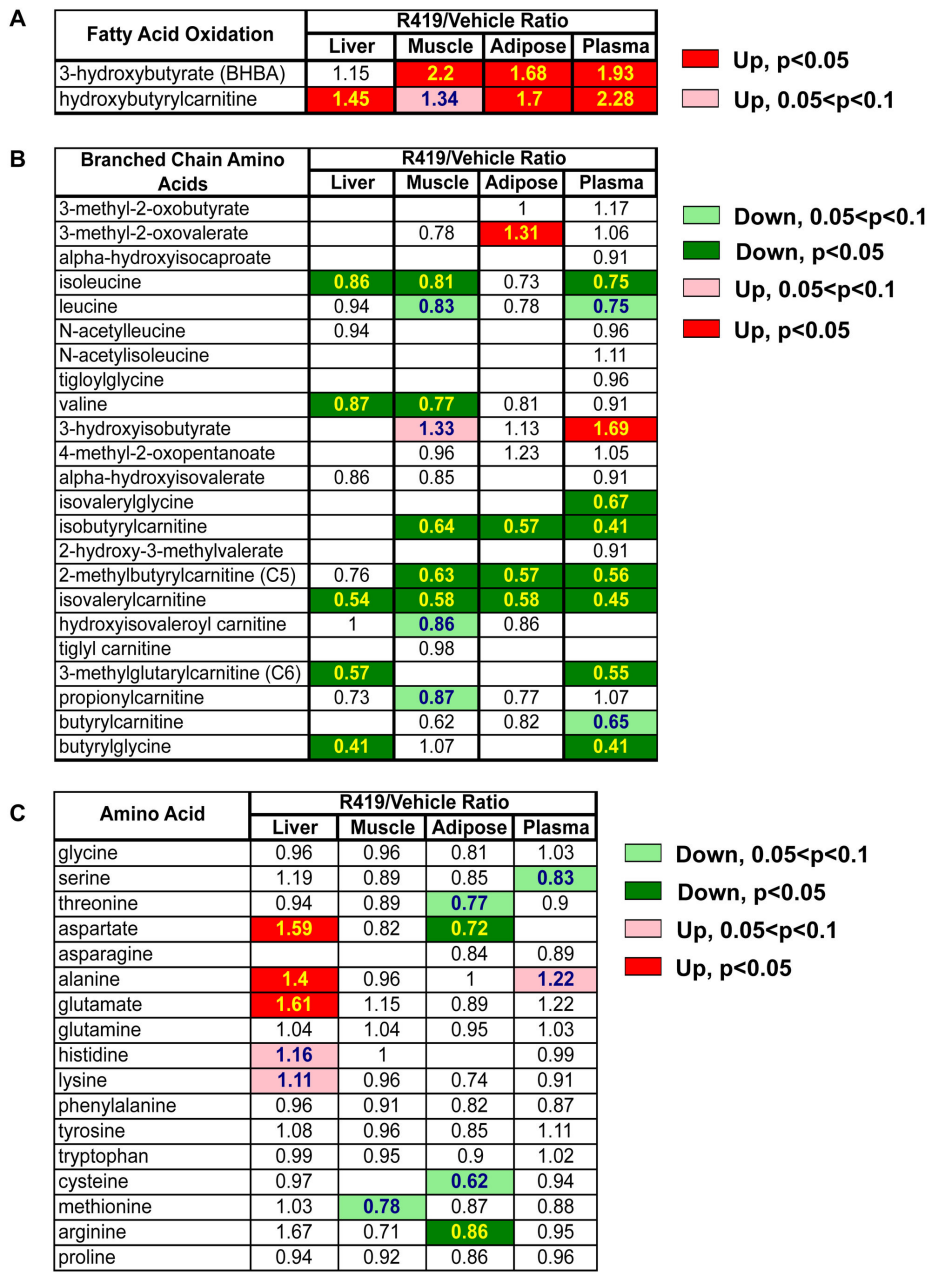
Effects on fatty acid, BCAA, and amino acid pathways in *db/db* mice treated with R419. Male *db/db* mice (8 weeks old) were PO QD dosed with vehicle or 10 mg/kg R419. Thirty minutes after oral dosing, liver, muscle, and adipose tissues and plasma were collected at 3 days (n = 6) of compound administration and analyzed at Metabolon for metabolite profiles. *A*: Heat map of fatty acid oxidation metabolites in liver, muscle, adipose, and plasma from R419-treated mice shown relative to vehicle. *B*: Heat map of intermediates of branched chain amino acid catabolism in liver, muscle, adipose, and plasma from R419-treated mice shown relative to vehicle. *C*: Heat map of amino acids in liver, muscle, adipose, and plasma from R419-treated mice shown relative to vehicle. Biochemical data were analyzed using Welch’s two-sample t-tests.

In muscle, fat, and plasma, R419-treatment resulted in a significant elevation of 3-hydroxybutyrate (BHBA) (Figure 5A), a ketone body which can be produced from acetyl CoA generated via mitochondrial fatty acid breakdown, and is consistent with the role of AMPK in regulating fatty acid β-oxidation [32]. In the liver, no difference was apparent in BHBA levels between R419-treated animals compared to the vehicle. However, given that the liver is the primary site of ketone body synthesis [33], the marked increase in the plasma was likely due to increased liver BHBA production, followed by secretion into the plasma. Levels of hydroxybutyrylcarnitine, the carnitine-modified form of BHBA [34], were similarly increased in all compartments sampled. 

One novel finding was a significant decrease in intermediates of the branched chain amino acids (BCAA), which are metabolized in the mitochondria into products that feed into the TCA cycle. Marked reductions in intermediates representing the catabolic pathways for all three BCAA were observed across all three major metabolic organs and in plasma with R419 treatment (Figure 5B). This cross-compartment decrease mediated by R419 appeared specific for the BCAA pathway since relative levels of the other amino acids were not similarly affected (Figure 5C). We also observed a reduction in the relative levels of butyrylglycine and butyrylcarnitine in liver and plasma. These metabolites can be derived from fatty acid β-oxidation but they can also be produced via an alternate “R” pathway of isoleucine catabolism. This pathway is limited under normal conditions but can increase when upstream intermediates accumulate during impaired S pathway function [35,36]. The overall decrease in tissue BCAA in conjunction with the decrease in plasma isoleucine, leucine, and additional BCAA intermediates is consistent with increased BCAA catabolism in the mitochondria and suggests that mitochondrial function is improved by R419.

### R419 treatment increases palmitate oxidation in liver, skeletal muscle, and adipose tissue

**Figure 6:**
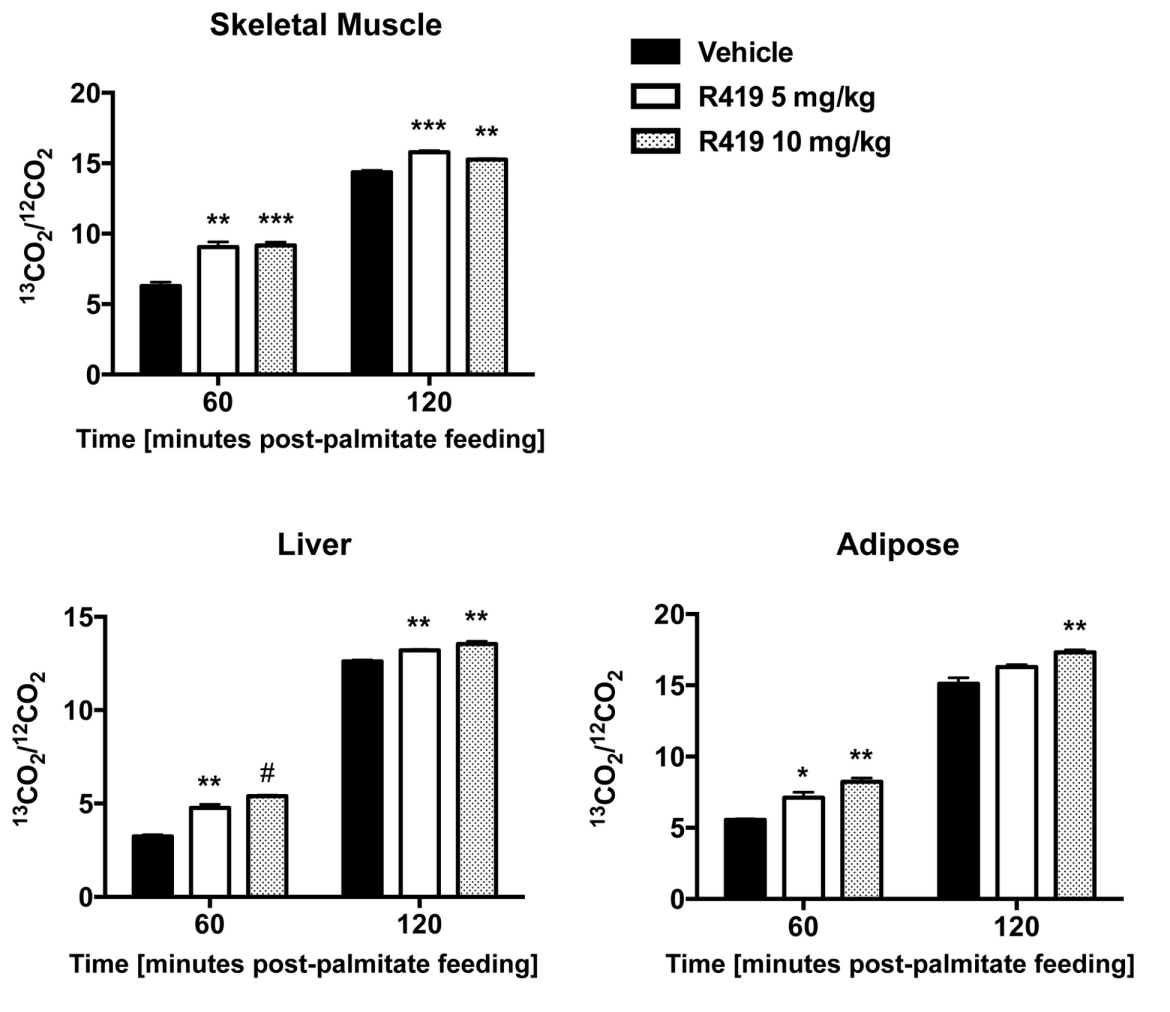
Increased palmitate oxidation in *db/db* mice treated with R419. Male *db/db* mice (8 weeks old) were PO QD dosed with vehicle, 5 mg/kg R419, or 10 mg/kg R419 for 8 days. On day 8, mice were orally gavaged with 0.5 mg/kg [U-^13^C]-palmitate 30 minutes after compound dosing. Liver, skeletal muscle, and adipose samples were collected at 60 and 120 minutes following palmitate administration (n=4). Complete oxidation of [U-^13^C]-palmitate to ^13^CO_2_ is shown as ^13^CO_2_ enrichment (^13^CO_2_/^12^CO_2_ ratio) in skeletal muscle, liver, and adipose. The data are presented as mean (bar) ± SEM (line). Statistical analyses between an R419-treated group and the corresponding vehicle control were performed using the unpaired 2-tailed Student *t* test. Asterisks *, **, *** and # represent p < 0.05, p < 0.01, p <0.001, and p < 0.0001, respectively for R419 treatment compared to vehicle.

The elevated levels of BHBA detected by the global metabolite profiling are an indirect indicator that R419 treatment increases lipid oxidation. In order to determine whether R419 had a direct effect on β-oxidation, [U-^13^C]-palmitate was orally administered to non-fasted mice treated with either vehicle or R419 for one week followed by measurements of ^13^CO_2_ to ^12^CO_2_ ratio in various tissues after palmitate feeding. Appearance of ^13^CO_2_ occurs via β-oxidation of the labeled palmitate to release ^13^C-acetyl CoA, which must pass through one round of the TCA cycle before the labeled carbon can be given off as ^13^CO_2_. An increased β-oxidation rate will result in a corresponding increase in ^13^CO_2_ enrichment. In agreement with the elevation in relative BHBA levels detected in liver, adipose, and skeletal muscle tissues, an increase in ^13^CO_2_ enrichment is observed for R419 treatment across all three tissues at both 60 and 120 minutes after palmitate administration. Ratios of ^13^CO_2_ to ^12^CO_2_ were higher for all treatment groups, including the vehicle, at 120 minutes compared to 60 minutes after palmitate feeding (Figure 6). This time-dependent elevation was due to the slow absorption kinetics of the orally administered tracer suspension [37]. In contrast to the liver and adipose tissue, no dose-dependence in ^13^CO_2_ enrichment was evident in skeletal muscle. All the data together demonstrate that chronic R419 treatment of *db/db* mice directly upregulated fatty acid oxidation in liver, skeletal muscle, and adipose tissue. 

### R419 treatment increases glucose oxidation in liver, skeletal muscle, and adipose tissue

**Figure 7:**
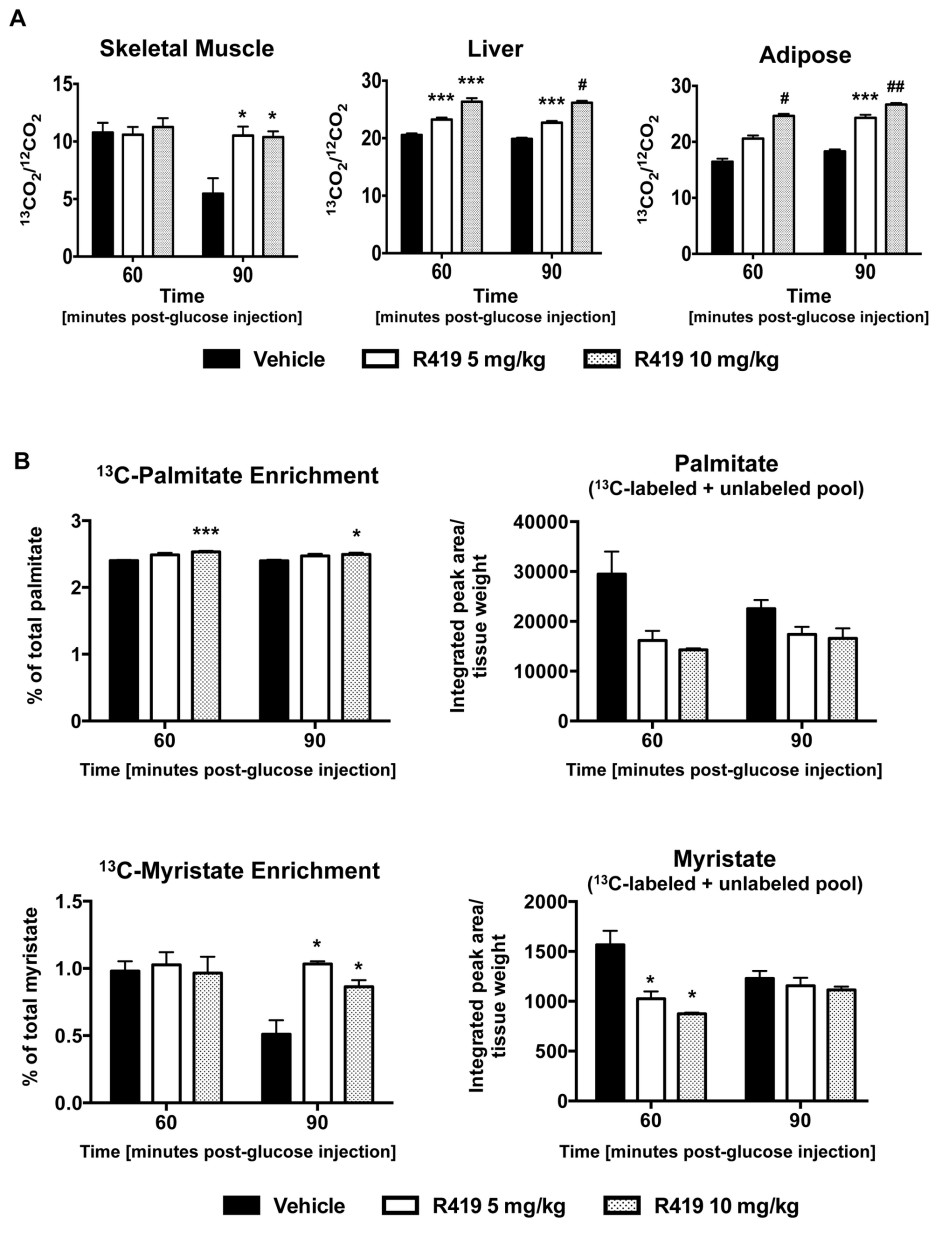
Effects on glucose metabolism in *db/db* mice treated with R419. Male *db/db* mice (8 weeks old) were PO QD dosed with vehicle, 5 mg/kg R419, or 10 mg/kg R419 for 8 days. On day 8, mice were given an intraperitoneal injection of 0.5 mg/kg [U-^13^C]-D-glucose 30 minutes after compound dosing. Liver, skeletal muscle, adipose, and plasma samples were collected at 60 and 90 minutes following glucose injection (n=4). *A*: ^13^CO_2_ enrichment (^13^CO_2_/^12^CO_2_ ratio) in skeletal muscle, liver, and adipose. *B*: ^13^C-labeled palmitate or myristate enrichment (% of palmitate or myristate) and normalized palmitate or myristate measured following saponification of skeletal muscle acylglycerols and acylcarnitines. % of total fatty acid was obtained using the following formula, (integrated peak area of ^13^C-labeled fatty acid)/ (integrated peak area of ^13^C-labeled plus unlabeled fatty acid). Total fatty acid (labeled plus unlabeled) was normalized by tissue weight. The data are presented as mean (bar) ± SEM (line). Statistical analyses between an R419-treated group and the corresponding vehicle control were performed using the unpaired 2-tailed Student *t* test. Asterisks *, ***, #, and ## represent p < 0.05, p <0.001, p < 0.0001, and p < 0.00001 respectively for R419 treatment compared to vehicle.

Despite the robust enhancement of glucose uptake and reduction in gluconeogenesis produced by R419 treatment *in vitro*, there were no dramatic effects on glucose metabolic pathways observed in the global metabolite profiling (data not shown). In order to better understand the *in vivo* effects of R419 on glucose metabolism, we utilized a more sensitive [U-^13^C]-D-glucose tracer analysis. Non-fasted db/db mice treated with vehicle or R419 for one week were given an intraperitoneal injection of a [U-^13^C]-D-glucose tracer (0.5 mg/kg) followed by measurements of tracer carbon incorporation into various metabolites after glucose injection. In both liver and adipose tissues from R419-treated mice, a clear dose-dependent increase in ^13^CO_2_ enrichment was observed at both 60 and 90 minutes post-tracer administration (Figure 7A). Skeletal muscle treated with R419 displayed a contrasting pattern of ^13^CO_2_ enrichment. At 60 minutes, no differences between vehicle and compound treatment were apparent but by 90 minutes, the enrichment in ^13^CO_2_ in the vehicle declined by 50% whereas the tracer oxidation was clearly sustained in the R419 treated animals. Similar to the palmitate tracer results, no dose-dependence in complete glucose tracer oxidation was observed in skeletal muscle, suggesting that muscle mitochondria are fully responsive to R419 treatment with the maximal effects reached at the lower dose. 

Measurements of skeletal muscle palmitate and myristate following saponification of muscle acylglycerols and acylcarnitines showed significant decreases in the levels of both these fatty acid pools in R419-treated mice (Figure 7B), and correlates well with the increased palmitate oxidation shown with the ^13^C-palmitate tracer (Figures 6). At both 60 and 90 minutes, the palmitate and myristate pools from R419-treated animals were significantly enriched in ^13^C-labeled fatty acids, with the myristate population containing a two-fold higher level of ^13^C-labeled myristate at the 90-minute timepoint in the R419-treated mice compared to vehicle. Oxidation of this pool of fatty acids enriched in ^13^C-labeled molecules may contribute to the sustained elevation in ^13^CO_2_ release observed in the skeletal muscle at 90 minutes with R419 treatment. These ^13^C-labeled fatty acids were muscle-derived as no increase in the ^13^C-labeled fraction of palmitate was observed in either the plasma or the liver (Figure S4). 

## Discussion

In this report, we have described in detail the effects of treatment with R419, a novel small molecule that inhibits complex I, resulting in potent stimulation of AMPK activity *in vitro* and *in vivo*, and have characterized its effects both in cell lines and in a mouse model of diabetes. Metabolic profiling experiments performed on *db/db* mice in order to better understand the changes in nutrient metabolism induced by short term R419 treatment revealed striking impacts on mitochondrial function such as upregulated fatty acid and glucose oxidation as well as BCAA catabolism in liver, muscle, and adipose tissue.

Using both mouse liver mitochondria and cultured cells, we systematically determined that R419 activated AMPK by perturbing cellular energy status, specifically via inhibition of respiratory complex I. *In vitro* complex I activity was also inhibited by metformin, as expected, albeit at higher concentrations. As shown previously for metformin [20], the observed suppression of cAMP-stimulated glucose production likely results from this decrease in cellular energy state produced by R419 inhibition of complex I, rather than its ability to activate AMPK, providing a rationale for the maintained suppression of glucose production in the AMPK α1/α2 KO hepatocytes by R419 (Figure 3C). Reduction of hepatic glucose production via suppression of gluconeogenesis is attributed as the primary effect of metformin in diabetes therapy [38-40]. Metformin has been shown to be a substrate of organic cation transporters (OCTs) and genetic studies have highlighted the importance of OCT1 in the clinical response to metformin therapy [41-45]. In contrast, cellular transport of R419 did not involve OCT1 (Figure S5) and suggests that R419 may be a therapeutic alternative for diabetic individuals who carry reduced function OCT1 polymorphisms. 

Although we observed R419-mediated suppression of gluconeogenesis *in vitro*, we did not see any dramatic changes in liver glucose pathways in the global metabolite analysis, which could possibly be due to sample collection during the fed state where hepatic glucose production rates would be lower due to insulin action. One novel result revealed by the metabolite analysis was the decrease across all sampled compartments in intermediates of BCAA catabolism, which could be particularly significant as numerous studies demonstrate a strong positive correlation of BCAA-related metabolite clusters with metabolic disease. The finding that relative levels of plasma BCAA metabolites were also reduced could potentially provide valuable biomarkers for assessing efficacy of R419 in clinical studies. Metabolomics studies in humans have shown that BCAA-related metabolites are strongly associated with insulin resistance [46-48], and can be predictive of improvement in insulin sensitivity with intervention [48,49]. Additionally, there is evidence that changes in BCAA levels may correlate with the relative efficacy of interventions for controlling glucose homeostasis [48,50]. High concentrations of BCAA, which are catabolized by the mitochondria, may decrease the efficiency of glucose and fatty acid oxidation. The consequent accumulation of incompletely oxidized substrates as well as increased mitochondrial stress may eventually result in impaired insulin function and glucose intolerance. 

In our mouse diabetes model, we observed significant decreases in BCAA intermediates in liver, muscle and adipose tissues, as well as plasma, which strongly suggest that mitochondrial function is improved with R419 treatment and could contribute to the improvements in glucose and insulin tolerance observed in R419-treated *db/db* mice. Whether this effect on BCAA catabolism is due to the mitochondrial perturbation or is a direct effect of AMPK activation is unclear as no direct links between AMPK and BCAA catabolism have been reported. Mice overexpressing a kinase-dead mutant of AMPK α2 specifically in skeletal muscle did not display insulin resistance under normal conditions or when challenged with high fat diet [51] which suggests that deleting AMPK activity selectively in muscle is not sufficient to impact BCAA catabolism. Alternatively, it is possible that the relationship between AMPK, BCAA catabolism, and insulin responsiveness has not been examined under optimal dietary conditions. Indeed, previous studies have shown that the effects of BCAA on insulin resistance in rats are more pronounced in the context of HFD feeding [48]. Further animal studies performed in a dietary background where elevated BCAA are particularly deleterious may reveal additional therapeutic benefits from the effect of R419 on this pathway. 

A significant elevation in BHBA, an indicator of fatty acid oxidation, was seen in muscle, fat, and plasma of R419-treated mice. BHBA was recently identified as an endogenous inhibitor of class I histone deacetylases (HDACs), specifically enhancing HDAC acetylation and expression of the genes encoding oxidative stress resistance factors forkhead box protein O3A (FOXO3A) and metallothionien 2 (MT2) [52]. Furthermore, class I–specific HDAC inhibition by the small molecule MS275 in *db/db* mice increased expression of peroxisome proliferator-activated receptor gamma coactivator 1-alpha (PGC-1α, mitochondrial biogenesis, and oxygen consumption [53]. These reports raise the possibility of BHBA being an important bioactive molecule that induces mitochondrial biogenesis and reduces oxidative stress. 

In order to confirm and also expand upon the findings from the *in vivo* metabolite profiling, we utilized ^13^C-palmitate and ^13^C-glucose tracer analyses to determine the metabolic fate of palmitate and glucose upon entry into liver, adipose tissue, and skeletal muscle of *db/db* mice and found that R419 increased ^13^CO_2_ release in all three tissues, indicating that direct and indirect oxidation of both fatty acid and glucose via the TCA cycle was increased. Since inhibition of complex I by R419 leads to reduction of the oxygen consumption rate *in vitro*, it may seem paradoxical that substrate oxidation to CO_2_ by the TCA cycle is increased without a corresponding increase in energy demand. However, the reduction in respiration observed in R419-treated mitochondria can be completely rescued by adding succinate, which bypasses the complex I block by providing reducing equivalents through complex II. Succinate is one of the end products of valine/isoleucine catabolism and also β-oxidation of fatty acids containing an odd number of carbons. These are both processes that are upregulated in *db/db* mice by R419 treatment. In addition, the reduced flavin adenine dinucleotide (FADH_2_) produced during the first step of fatty acid β-oxidation can supply electrons to the respiratory chain downstream of complex I via an electron-transferring flavoprotein (ETF). These effects observed *in vivo* for R419, similar to the impact of succinate addition *in vitro*, may attenuate the suppressed respiration that occurs secondary to complex I inhibition. 

**Figure 8:**
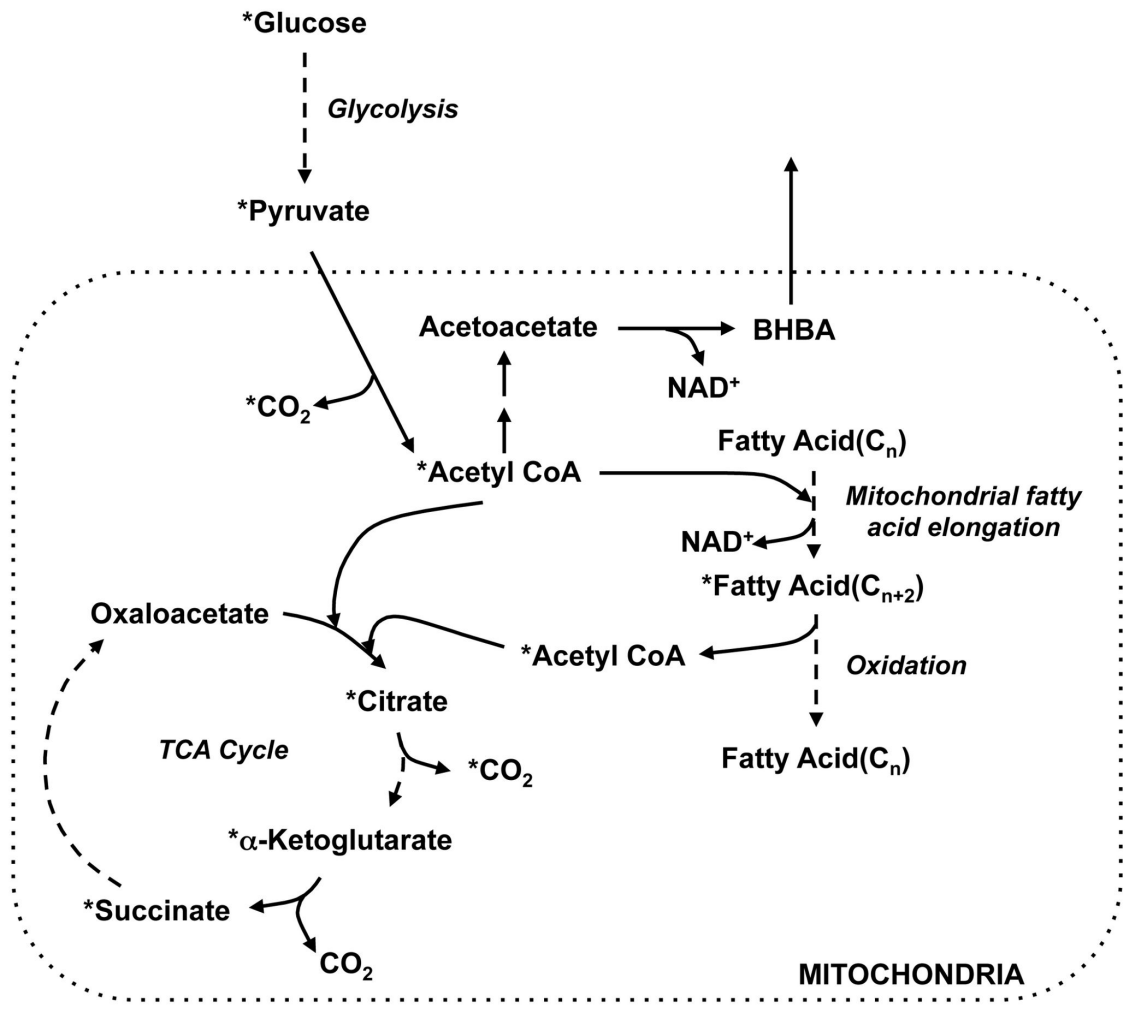
Scheme illustrating possible metabolic route of ^13^C-glucose to ^13^CO_2_ occurring in skeletal muscle from R419-treated *db/db* mice. Input glucose is broken down via glycolysis to pyruvate, which is decarboxylated to CO_2_ and acetyl CoA. ^13^C-acetyl CoA can be condensed with oxaloacetate to form citrate, used to elongate short-chain fatty acids or routed for BHBA production. Skeletal muscle mitochondrial short chain fatty acid elongation utilizes acetyl-CoA acyltransferase, 3-hydroxyacyl-CoA dehydrogenase, enoyl-CoA hydratase, enoyl-CoA reductase, and NADH as a reducing agent. Although in general, R419-treatment resulted in reduction of palmitate and myristate levels in the skeletal muscle, these fatty acid pools were enriched in ^13^C-labeled palmitate and myristate. ^13^C-palmitate and ^13^C-myristate can be broken down by β-oxidation, releasing ^13^C-acetyl CoA, which can enter the TCA cycle and be released as ^13^CO_2_ by either isocitrate dehydrogenase or α-ketoglutarate dehydrogenase after multiple cycles. Asterisks indicate a molecule is ^13^C-labeled. Dashed arrows indicate multistep conversions.

Since NAD^+^ is an obligatory cofactor for glucose, fatty acid, and BCAA catabolism, compensatory pathways must be activated for circumventing R419 inhibition of NADH oxidation by complex I in order to sustain nutrient breakdown until metabolic homeostasis is recovered. Our metabolomics and tracer studies provided some insights into how this compensation was achieved (Figure 8). As revealed in the global metabolite profiling, R419 significantly increased BHBA production in skeletal muscle, adipose, and plasma, indicating that some of the acetyl CoA generated from pyruvate, fatty acid, and BCAA breakdown is routed to ketone production, which enables mitochondrial NAD^+^ generation through conversion of acetoacetate to BHBA. In the skeletal muscle, according to the ^13^C glucose tracer studies, the ^13^C-labeled fractions of myristate and palmitate were significantly elevated by R419 treatment despite the reduction in total pools of the two fatty acids, a pattern that is unique to the muscle and not observed in liver or adipose tissue (data not shown). This increase in fatty acid ^13^C-labeling may have contributed to the sustained release of ^13^CO_2_ observed at 90 minutes in skeletal muscle from R419-treated animals, a timepoint where glucose oxidation drops in the vehicle by ~50% and is also a pattern unique to skeletal muscle. At this timepoint, release of the labeled carbons derived from the glucose tracer as CO_2_ would be due to β-oxidation of the ^13^C-enriched fatty acids. These data are consistent with a model in which the excess acetyl-CoA produced from the breakdown of pyruvate, fatty acid, and likely also BCAA induced by R419 is absorbed by the fatty acid pool, via mitochondrial fatty acid elongation [54], and eventually released as CO_2_ (Figure 8). Indeed, isotopomer data (not shown) supports a model where oxaloacetate levels are increased through pyruvate carboxylase, which could sustain the continued absorption of acetyl-CoA generated from fatty acid at 90 minutes, allowing the tracer carbons to be released as CO_2_ through oxidation in the TCA cycle. Similar to BHBA production, this effect on routing of ^13^C-labeled carbons from the glucose tracer likely reflects an alternate method of NAD^+^ regeneration since mitochondrial fatty acid elongation utilizes NADH. 

In order to assess R419 effects on substrate utilization in an isolated system, we performed both glucose and fatty acid oxidation studies using different cell culture models. Similar to our *in vivo* experiments, analysis of [1,2-^13^C_2_]-D-glucose and [U-^13^C]-palmitate tracer metabolism in primary human skeletal muscle cells treated with R419 also yielded a dose-dependent enrichment in ^13^CO_2_ produced for each tracer (Figure S6A and B) and [1-^14^C]palmitate oxidation into acid-soluble metabolites was increased in primary hepatocytes as shown in Figure 4A. These data indicate that R419 increases substrate oxidation of both fatty acids and glucose in an isolated *in vitro* system. 

Of note, we also extended our *in vitro* studies using HepG2 hepatocarcinoma cells and C2C12 mouse myotubes and found that complete fatty acid oxidation assayed using production of ^3^H-labeled water from oxidation of [9,10-^3^H(N)]-oleate was reduced about 10-20% by R419, suggesting that some variability occurs in R419-induced fatty acid oxidation *in vitro*. It has been reported that some cultured cell lines may be impaired in their capacity to oxidize long chain fatty acids which may also contribute to the variability in R419 responses observed [55]. In addition, although the core biochemical pathways may be identical across cells, responses to the metabolic alteration triggered by R419 can be context-dependent and influenced by factors such as nutrient availability in the medium and also the specific functions of the tissue of origin.

Several differences should be considered when comparing the effects observed when cells are treated *in vitro* to when mice are treated with R419. R419 exposure will be variable *in vivo* due to the rapid elimination of the compound (Figure S2), in contrast to the constant *in vitro* exposure. Based upon the plasma exposure profile, R419 administration to mice at doses that affect nutrient metabolism would result in a rapid but transient inhibition of complex I, followed by a rapid return to baseline. The glucose and palmitate tracer data were collected at timepoints ranging from 90 to 150 minutes after R419 delivery, which is during the recovery period so these results likely reflect the physiological return to metabolic homeostasis. 

Since our metabolite profiling and tracer studies suggested that R419 increased mitochondrial function in *db/db* mice, we performed preliminary experiments comparing the efficacy of R419 in the same model alongside metformin as a reference compound since it is the first line therapy used in T2D and is also a complex I inhibitor (Figure S7). Improvements in glucose handling, fasting glucose, and also fasting insulin comparable to metformin were observed in *db/db* mice orally dosed once daily for 11 days with R419. In an aged diet-induced obesity (DIO) model (on high fat diet for 39 weeks), liver triglyceride content was reduced with R419 treatment (Figure S8), which correlates with the effects on lipid metabolism identified in the *db/db* studies. Of note, no statistically significant impact on either food intake or body weight gain was observed in animals treated with R419 in the two studies (data not shown). Currently, further studies are ongoing to validate these preliminary findings and to better understand how R419 affects glucose and lipid homeostasis *in vivo*.

In conclusion, by performing a comprehensive analysis with R419, a novel complex I inhibitor, we demonstrated the potential physiological consequences, as well as therapeutic benefits, of regulating mitochondrial function in diabetic models. Molecules with the properties of R419 may ameliorate debilitating conditions such type 2 diabetes and its complications. 

## Supporting Information

Figure S1 **GLUT4 promoter activation by R419.** C2C12 myotubes were transfected with the GLUT4 promoter and the control promoter constructs (LightSwitch Dual Assay System, SwitchGear genomics, CA) according to the manufacturer’s instruction. At 24 hours after transfection, the cells were incubated with R419 or AICAR overnight. Luciferase activity of the GLUT4 promoter and the control promoter was measured with LightSwitch assay reagents using a SpectraMax luminometer. The data are normalized against luciferase activity of the control promoter and presented as mean ± SEM (n=3). Ordinary one-way ANOVA with the Dunnett ad-hoc test was performed, and the multiple comparison test was done against the DMSO control. Asterisk ** represents p < 0.01.
(TIF)

Figure S2 ***Invivo* AMPK activation by a single oral dose of R419 in C57BL6 Mice.** Male C57BL/6J (9 weeks old) mice dosed orally with vehicle, R419 5 mg/kg, or R419 10 mg/kg were sacrificed at various timepoints after dosing. Liver and gastrocnemius muscle were collected for each timepoint. Lysates for western blotting were prepared by homogenizing frozen tissues in CST lysis buffer (Cell Signaling Technologies) containing complete protease inhibitor cocktail and PhosStop phosphate inhibitor (Roche Applied Sciences) using a tissue homogenizer. Lysates were normalized for protein concentrations prior to SDS-PAGE gel electrophoresis using the Pierce BCA kit (ThermoFisher Scientific). *A*: AMPK activation by R419 in skeletal muscle (gastrocnemius) and liver. Phosphorylation of AMPK and its substrates ACC and ULK1 in muscle and liver at 30 minutes post dose was examined by western blotting. Each lane represents the sample from each mouse. Actin was used for a loading control. *B*: ImageJ quantitation of muscle and liver AMPK activation at various time points following a single oral dose of R419. The densities of pAMPK signals on exposed films were scanned using ImageJ 64 (http://imageJ.nih.gov/ij), and normalized to those of actin. The data were further normalized to AMPK activity in vehicle control and are presented as mean (bar) ± SEM (line) (n=3). Ordinary two-way ANOVA with the Dunnett ad-hoc test was performed, and the multiple comparison test was done against each vehicle control. Asterisks *, ** and *** represent p < 0.05, p < 0.01 and p < 0.001, respectively.
(TIF)

Figure S3 **Pharmacokinetic properties of R419.** *A*: Pharmacokinetic profile in plasma after single dose administration by oral gavage to male C57BL/6 mice at 5 (squares) and 10 mg/kg (circles) of R419. Plasma concentration of the compound at specified time points was quantified by LC/MS/MS. The data are presented as mean (symbol) ± SEM (line) (n=3). Statistical analyses were not performed for this data set *B*: Area under the curve measurements corresponding to R419 dosing in (A). *C*: R419 pharmacokinetic parameters in male C57BL/6 mice after a single dose administration by oral gavage at 5 mg/kg or intravenously at 1 mg/kg.
(TIF)

Figure S4 **Effects on glucose metabolism in *db/db* mice treated with R419.** [U-^13^C]-D-glucose flux experiment was performed as described in Figure 7. Liver and plasma samples were collected following tracer glucose injection (n = 4). *A*: ^13^C-labeled palmitate enrichment (% of total palmitate) in plasma. *B*: ^13^C-labeled palmitate enrichment (% of total palmitate) and normalized total palmitate in liver. The data are presented as mean (bar) ± SEM (line). Statistical analyses between an R419-treated group and the corresponding vehicle control were performed using the unpaired 2-tailed Student *t* test. The asterisks ** represent p < 0.01 for R419 treatment compared to vehicle.
(TIF)

Figure S5 **OCT1-independent permeation of R419.** R419 was tested for OCT1-dependent compound permeation using MDCK-II cells over-expressing human organic cationic transporter 1 (OCT1 (+), open bar) and control green fluorescence protein (OCT1 (-), hatched bar) at Optivia Biotechnology Inc, Menlo Park, CA. The cells were incubated with 0.5 µM R419 for 5 minutes in the presence or absence of an OCT1 inhibitor, quinidine (100 µM). The compound in the cells was quantified by LC/MS/MS. The data are presented as mean (bar) ± SEM (line) of triplicate cultures. Ordinary two-way ANOVA with the Tukey ad-hoc test was performed. No statistical significance was reached between OCT1 (+) and (-) and between quinidine (+) and (-).
(TIF)

Figure S6 **Increased palmitate and glucose oxidation in human primary skeletal muscle cells *invitro*.** 5 x 10^5^ primary human skeletal muscle cells were differentiated for 5 days in DMEM/2% horse serum. Cells were washed with pre-warmed PBS and then incubated with differentiation medium containing either 50 µM [U-^13^C]-palmitate or 12.5 mM [1,2-^13^C_2_]-D-glucose (labeled glucose comprised 50% of total glucose in the medium) for one or 24 hours. Media containing tracer molecules also contained R419 (20 and 200 nM final concentrations). After the incubation, the media and cell pellets were collected and sent to SiDMAP, LLC (Los Angeles, CA) for isotope tracer analyses. Statistical analyses between an R419-treated group and the corresponding vehicle control were performed using the unpaired 2-tailed Student *t* test. Asterisks **, *** and # represent p < 0.01, p < 0.001 and p < 0.0001, respectively. The data are presented as mean (bar) ± SEM (line) for A: Palmitate oxidation in human skeletal muscle cells (n=3), and B: glucose oxidation in human skeletal muscle cells (n=6).
(TIF)

Figure S7 **R419 improves glucose tolerance in *db/db* mice.** Male *db/db* mice (8 weeks old) were PO QD dosed with vehicle, 5 mg/kg, or 10 mg/kg R419, or 200 mg/kg of metformin for 11 days. *A*: Oral glucose tolerance test (OGTT) following 11-day treatment (n=12/group). OGTT was performed 24 hours after the last dosing. Glucose was administered orally at 2 g/kg following a 6-hour fast with food removal at 5 AM. Repeated measures two-way ANOVA followed by the Dunnett ad-hoc test were performed and the multiple comparison test was done against the corresponding vehicle control at each timepoint. Significant variation among the treatment groups was not reached by repeated measures two-way ANOVA, however, Dunnett’s multiple comparison test showed significance between the R419 10 mg/kg group and the vehicle group at 30 and 60 minute timepoints and between the R419 5 mg/kg group and the vehicle group at 30 minute timepoint. *B*: Area under the curve corresponding to the graphs shown in A was calculated using GraphPad Prism version 6.0. Statistical significance between R419 and metformin treatment groups and the vehicle control was not reached (Ordinary one-way ANOVA with the Dunnett ad-hoc test, the multiple comparisons against the vehicle control). *C*: Fasting blood glucose levels following 11-day treatment (n=24/group). Blood sampling was performed 24 hours after the last dosing. Animals were fasted 6-hours prior to the blood sampling. Ordinary one-way ANOVA with the Dunnett ad-hoc test was performed for statistical analysis and the multiple comparison test was done against the vehicle control. *D*: Plasma insulin following an 11-day R419 treatment (n=12/group). Blood was collected 24 hours after the last compound dose, following a 6-hour fast. The data are presented as mean (symbol or bar) ± SEM (line). Ordinary one-way ANOVA with the Dunnett ad-hoc test was performed for statistical analyses and the multiple comparison test was done against the vehicle control. For all figures, asterisks *, and ** represent p < 0.05 and p < 0.01, respectively.
(TIF)

Figure S8 **Reduction of steatosis in aged diet-induced obese mice after treatment of R419.** Male C57BL6 mice were maintained on a high fat diet (HFD) since 6 weeks of age (diet-induced obesity, DIO). Treatments were started at the 39th week on HFD. R419 was prepared in HFD (D12492, Research Diet, Inc.). During the study, body weights and food intake were comparable between the HFD control and the R419 group. After 10 weeks of compound treatment, livers were harvested for triglyceride content and histological analysis *A*: Reduction of triglyceride content in liver from R419-treated aged DIO mice. The data are normalized to triglyceride content in aged DIO mice fed with control HFD. Unpaired two-tailed t-tests were performed between HFD control (n=8) and R419-treated group (n=8). Asterisk # represents p < 0.0001. *B*: Reduced hepatic steatosis in R419 treated mice. Frozen liver sections were stained with hematoxylin and eosin or oil red o dye, and examined by microscopy. Representative images for HFD control and R419 treated mice are shown at a 20 X magnification.
(TIF)

Table S1 **No clear inhibitory effects by R419 on activity of TCA enzymes *invitro*.** All the enzymes with the exception of complexes I, III, aconitase 2 and isocitrate dehydrogenases 2 and 3 were purchased from Sigma and tested according to protocols provided by the manufacturer. The rest of the enzyme activities were tested using mitochondrial lysate obtained by extracting purified mitochondria with two volumes of mammalian protein extraction reagent (Pierce) for 1 hour followed by a 10 min high speed spin in a table-top centrifuge. The assay conditions for complexes I, aconitase 2 and isocitrate dehydrogenases 2 and 3 were described elsewhere (S1-S5). Kinetics of NADH/NAD+ or NADPH/NADP^+^ transition was monitored spectrophotometrically at 340 nm. IC50s were presented as mean of multiple experiments (n≥2).
(TIF)

References S1 **All references cited in SUPPLEMENTAL MATERIALS are listed in a separate supporting information document.**
(DOCX)
