# Supplementary figures and images for: AMPK Activation through Mitochondrial Regulation Results in Increased Substrate Oxidation and Improved Metabolic Parameters in Models of Diabetes
Source: PLoS One. 2013 Dec 5;8(12):e81870. doi: 10.1371/journal.pone.0081870 (PMC3855387; doi:10.1371/journal.pone.0081870)

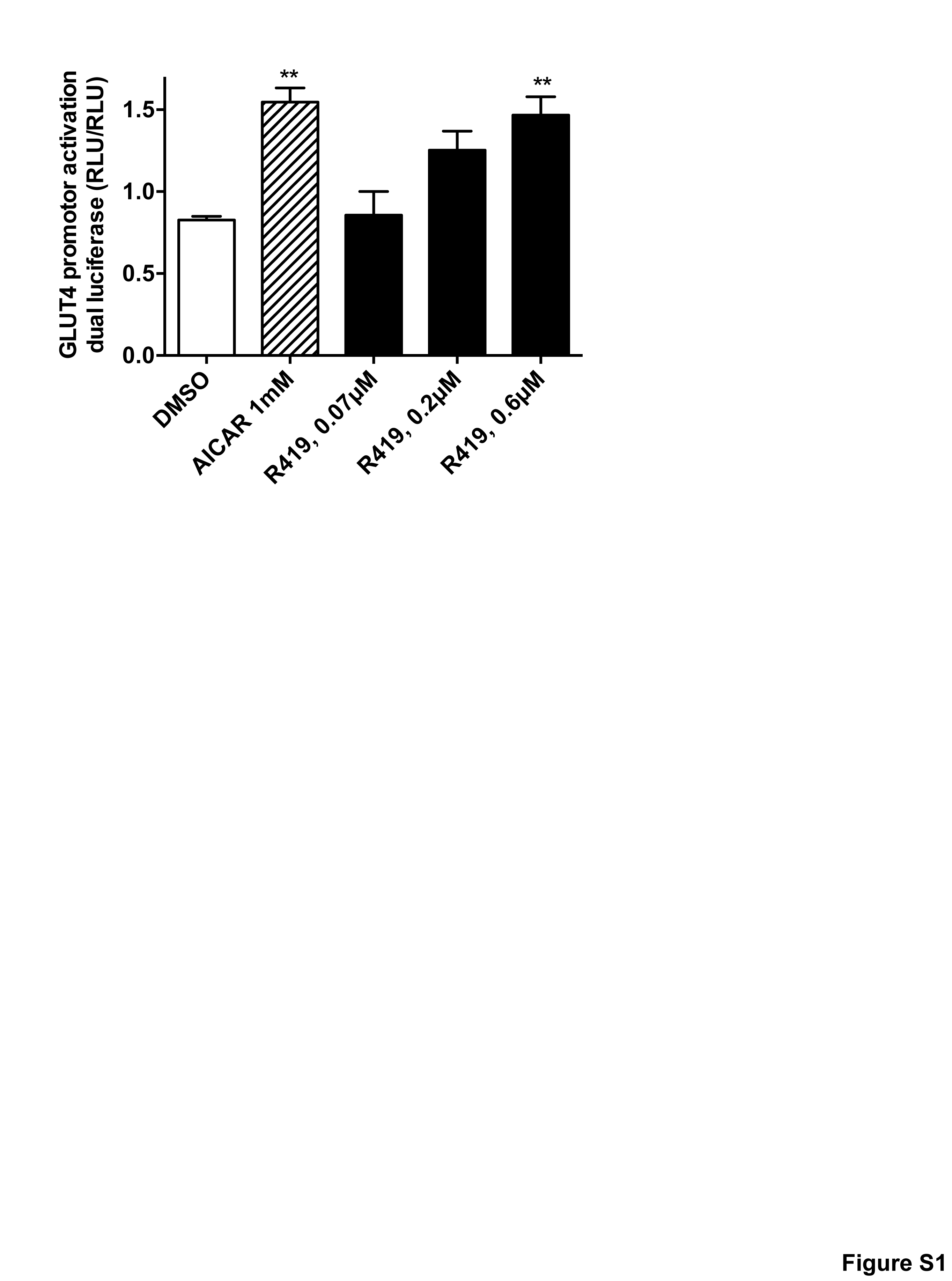

Supplement: Figure S1 — GLUT4 promoter activation by R419. C2C12 myotubes were transfected with the GLUT4 promoter and the control promoter constructs (LightSwitch Dual Assay System, SwitchGear genomics, CA) according to the manufacturer’s instruction. At 24 hours after transfection, the cells were incubated with R419 or AICAR overnight. Luciferase activity of the GLUT4 promoter and the control promoter was measured with LightSwitch assay reagents using a SpectraMax luminometer. The data are normalized against luciferase activity of the control promoter and presented as mean ± SEM (n=3). Ordinary one-way ANOVA with the Dunnett ad-hoc test was performed, and the multiple comparison test was done against the DMSO control. Asterisk ** represents p < 0.01. (TIF) [file pone.0081870.s001.tif]

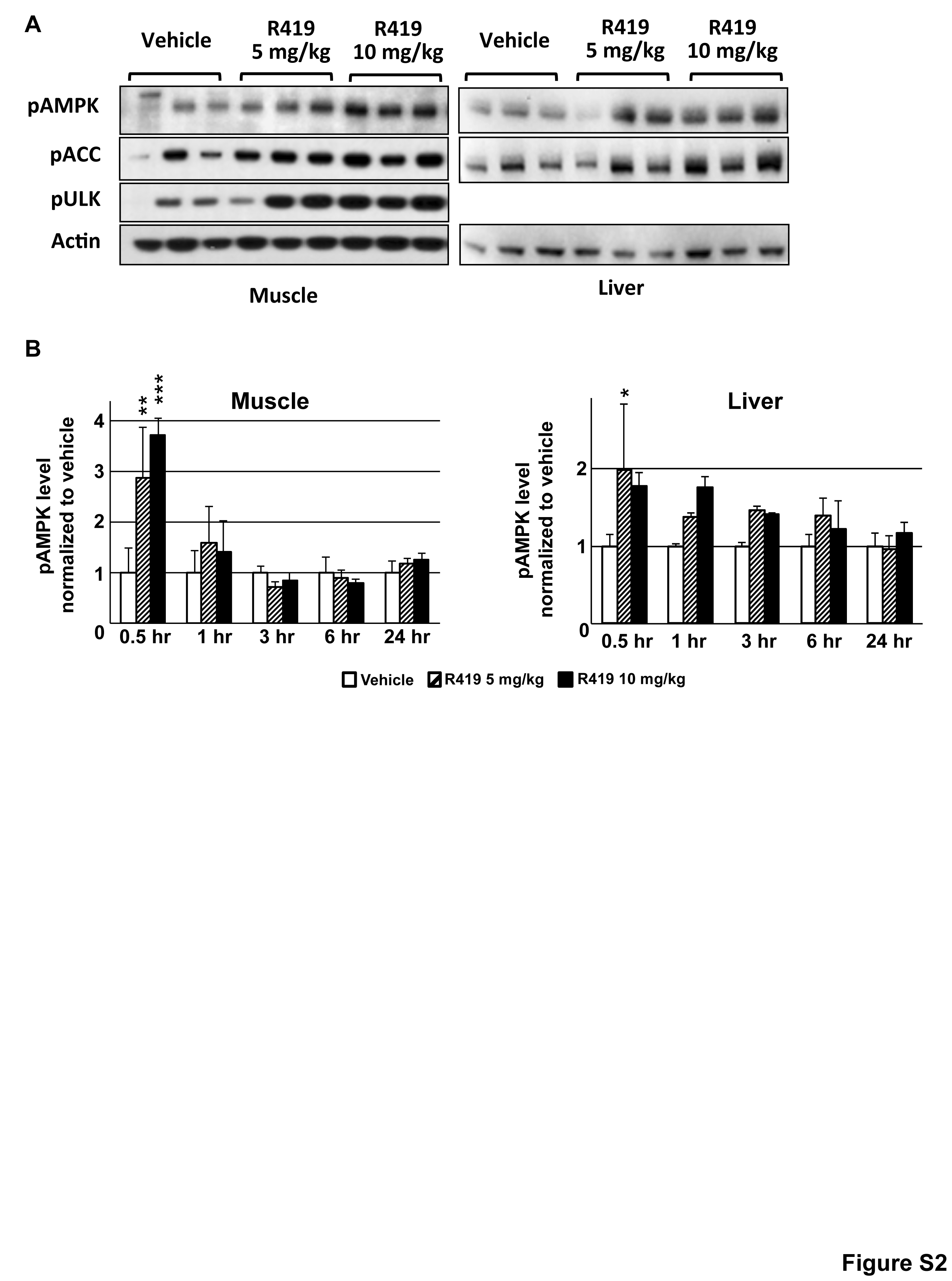

Supplement: Figure S2 — Invivo AMPK activation by a single oral dose of R419 in C57BL6 Mice. Male C57BL/6J (9 weeks old) mice dosed orally with vehicle, R419 5 mg/kg, or R419 10 mg/kg were sacrificed at various timepoints after dosing. Liver and gastrocnemius muscle were collected for each timepoint. Lysates for western blotting were prepared by homogenizing frozen tissues in CST lysis buffer (Cell Signaling Technologies) containing complete protease inhibitor cocktail and PhosStop phosphate inhibitor (Roche Applied Sciences) using a tissue homogenizer. Lysates were normalized for protein concentrations prior to SDS-PAGE gel electrophoresis using the Pierce BCA kit (ThermoFisher Scientific). A: AMPK activation by R419 in skeletal muscle (gastrocnemius) and liver. Phosphorylation of AMPK and its substrates ACC and ULK1 in muscle and liver at 30 minutes post dose was examined by western blotting. Each lane represents the sample from each mouse. Actin was used for a loading control. B: ImageJ quantitation of muscle and liver AMPK activation at various time points following a single oral dose of R419. The densities of pAMPK signals on exposed films were scanned using ImageJ 64 (http://imageJ.nih.gov/ij), and normalized to those of actin. The data were further normalized to AMPK activity in vehicle control and are presented as mean (bar) ± SEM (line) (n=3). Ordinary two-way ANOVA with the Dunnett ad-hoc test was performed, and the multiple comparison test was done against each vehicle control. Asterisks *, ** and *** represent p < 0.05, p < 0.01 and p < 0.001, respectively. (TIF) [file pone.0081870.s002.tif]

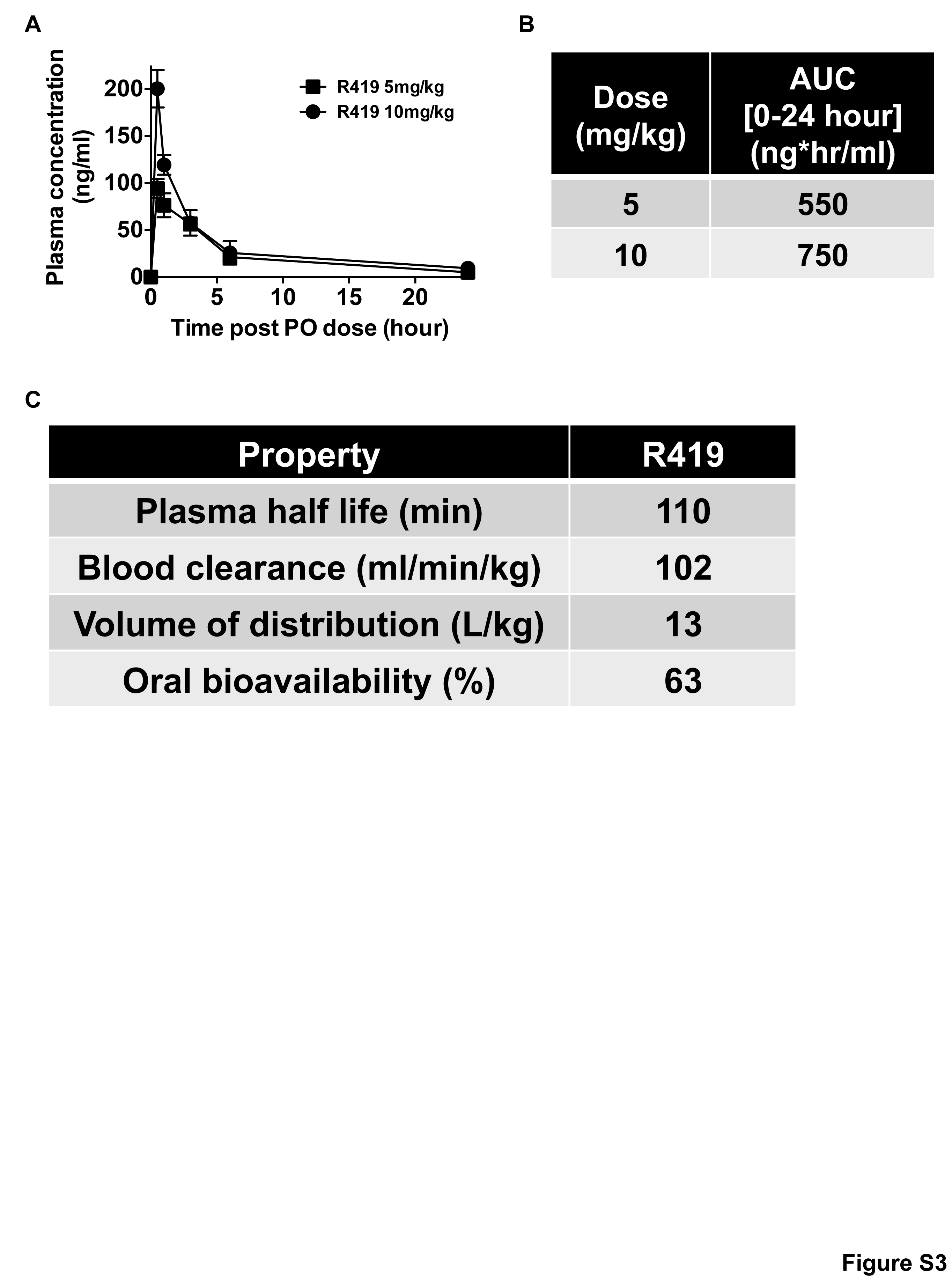

Supplement: Figure S3 — Pharmacokinetic properties of R419. A: Pharmacokinetic profile in plasma after single dose administration by oral gavage to male C57BL/6 mice at 5 (squares) and 10 mg/kg (circles) of R419. Plasma concentration of the compound at specified time points was quantified by LC/MS/MS. The data are presented as mean (symbol) ± SEM (line) (n=3). Statistical analyses were not performed for this data set B: Area under the curve measurements corresponding to R419 dosing in (A). C: R419 pharmacokinetic parameters in male C57BL/6 mice after a single dose administration by oral gavage at 5 mg/kg or intravenously at 1 mg/kg. (TIF) [file pone.0081870.s003.tif]

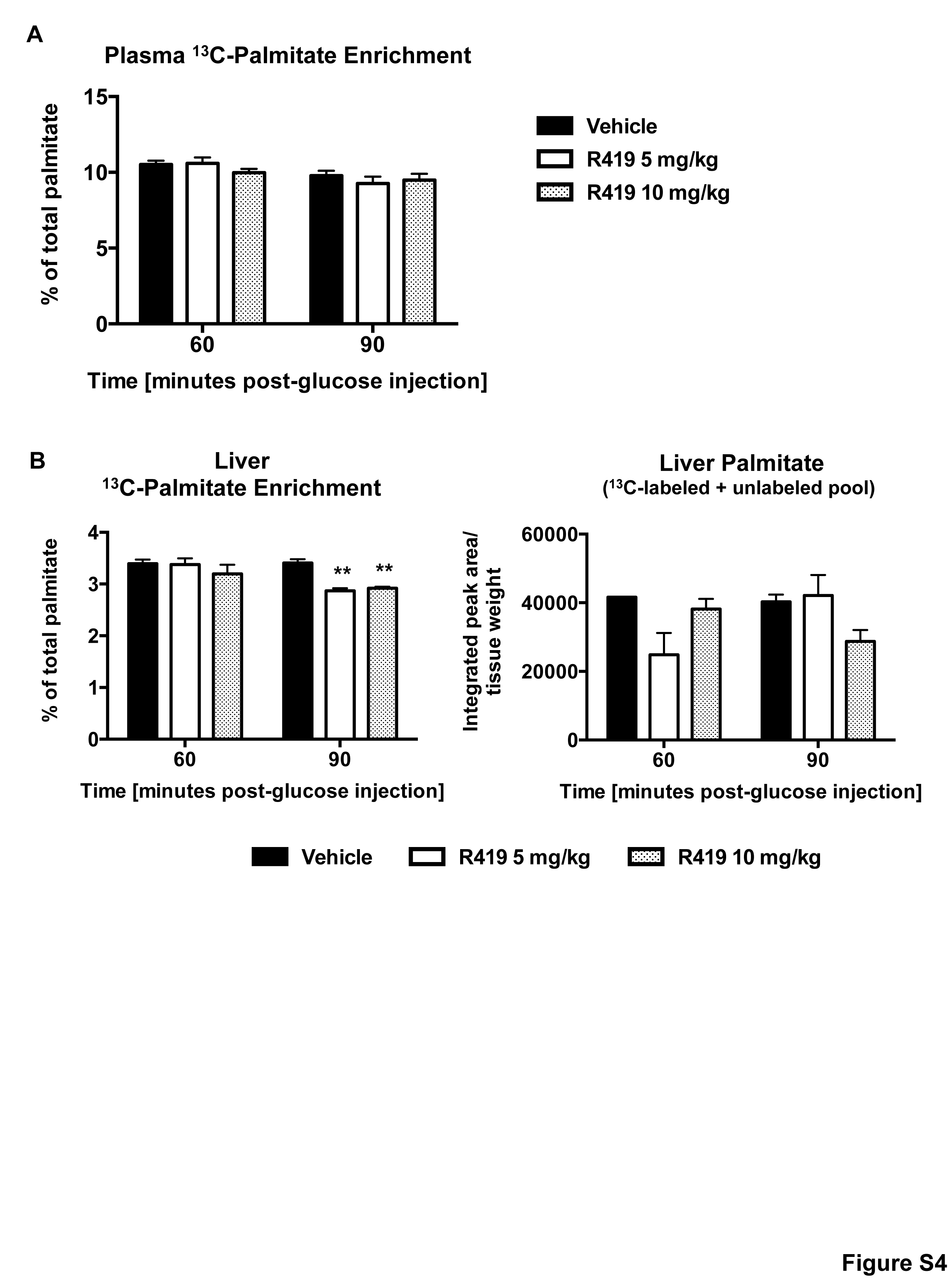

Supplement: Figure S4 — Effects on glucose metabolism in db/db mice treated with R419. [U-13C]-D-glucose flux experiment was performed as described in Figure 7. Liver and plasma samples were collected following tracer glucose injection (n = 4). A: 13C-labeled palmitate enrichment (% of total palmitate) in plasma. B: 13C-labeled palmitate enrichment (% of total palmitate) and normalized total palmitate in liver. The data are presented as mean (bar) ± SEM (line). Statistical analyses between an R419-treated group and the corresponding vehicle control were performed using the unpaired 2-tailed Student t test. The asterisks ** represent p < 0.01 for R419 treatment compared to vehicle. (TIF) [file pone.0081870.s004.tif]

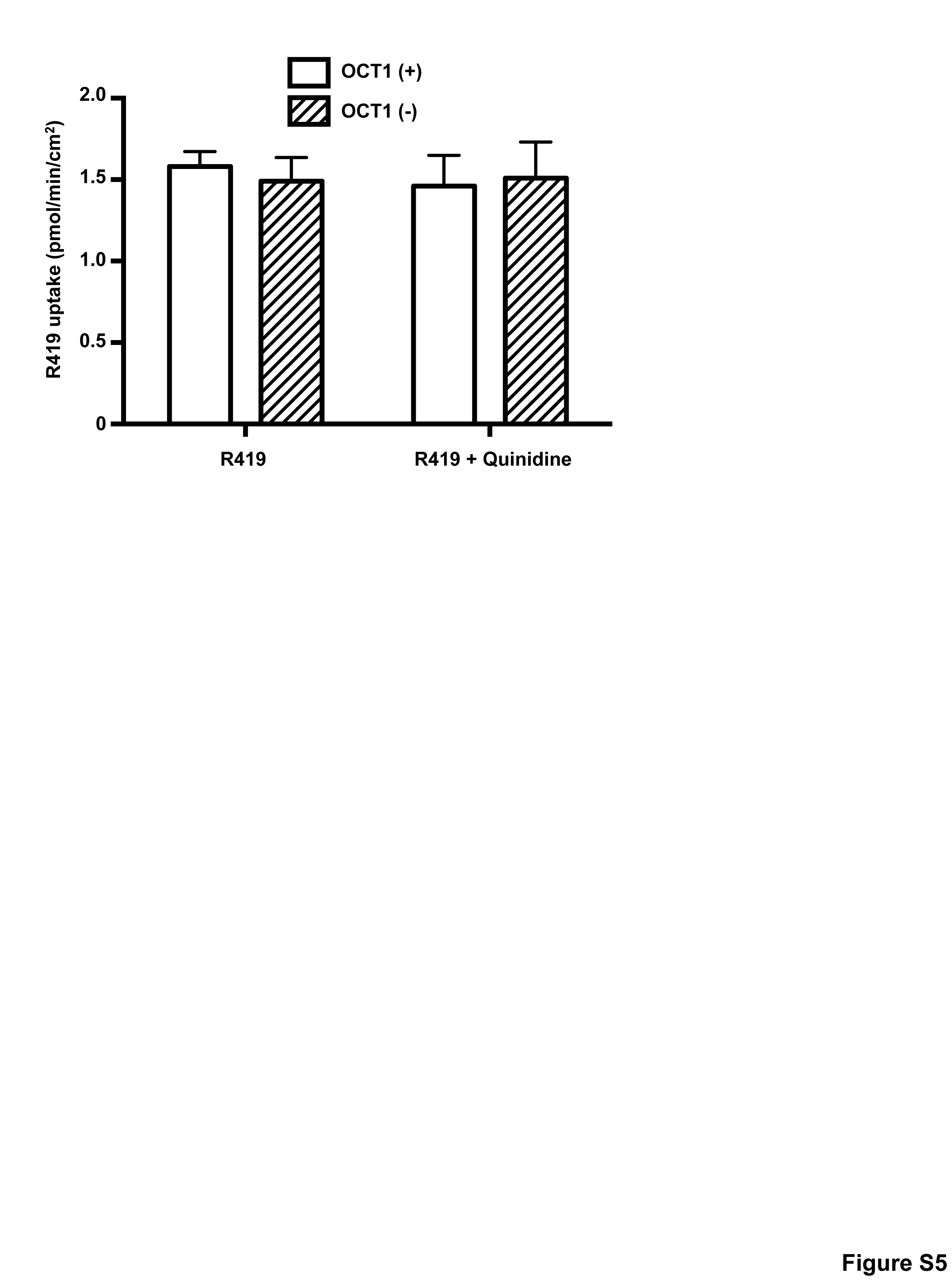

Supplement: Figure S5 — OCT1-independent permeation of R419. R419 was tested for OCT1-dependent compound permeation using MDCK-II cells over-expressing human organic cationic transporter 1 (OCT1 (+), open bar) and control green fluorescence protein (OCT1 (-), hatched bar) at Optivia Biotechnology Inc, Menlo Park, CA. The cells were incubated with 0.5 µM R419 for 5 minutes in the presence or absence of an OCT1 inhibitor, quinidine (100 µM). The compound in the cells was quantified by LC/MS/MS. The data are presented as mean (bar) ± SEM (line) of triplicate cultures. Ordinary two-way ANOVA with the Tukey ad-hoc test was performed. No statistical significance was reached between OCT1 (+) and (-) and between quinidine (+) and (-). (TIF) [file pone.0081870.s005.tif]

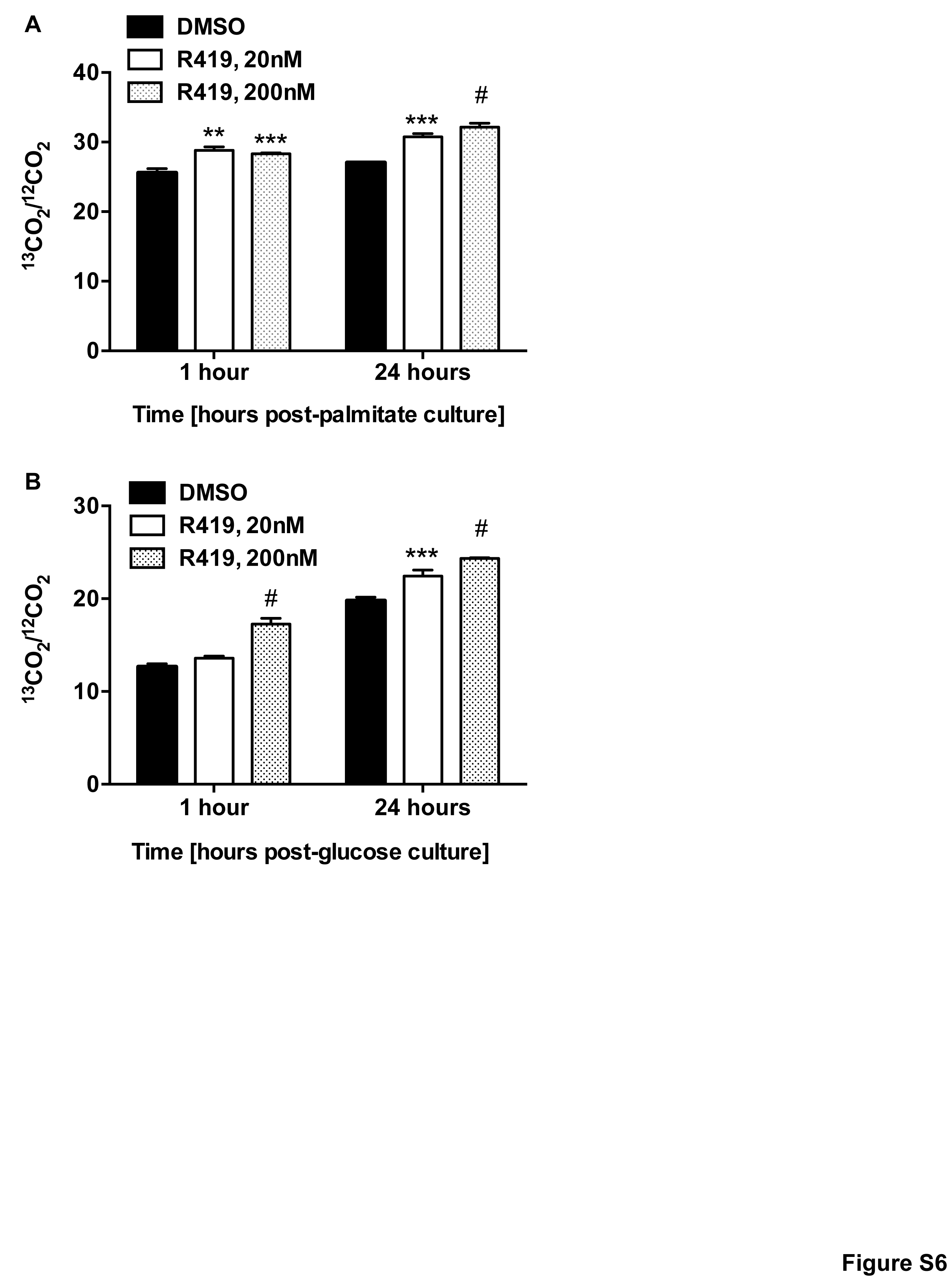

Supplement: Figure S6 — Increased palmitate and glucose oxidation in human primary skeletal muscle cells invitro. 5 x 10^5^ primary human skeletal muscle cells were differentiated for 5 days in DMEM/2% horse serum. Cells were washed with pre-warmed PBS and then incubated with differentiation medium containing either 50 µM [U-13C]-palmitate or 12.5 mM [1,2-13C2]-D-glucose (labeled glucose comprised 50% of total glucose in the medium) for one or 24 hours. Media containing tracer molecules also contained R419 (20 and 200 nM final concentrations). After the incubation, the media and cell pellets were collected and sent to SiDMAP, LLC (Los Angeles, CA) for isotope tracer analyses. Statistical analyses between an R419-treated group and the corresponding vehicle control were performed using the unpaired 2-tailed Student t test. Asterisks **, *** and # represent p < 0.01, p < 0.001 and p < 0.0001, respectively. The data are presented as mean (bar) ± SEM (line) for A: Palmitate oxidation in human skeletal muscle cells (n=3), and B: glucose oxidation in human skeletal muscle cells (n=6). (TIF) [file pone.0081870.s006.tif]

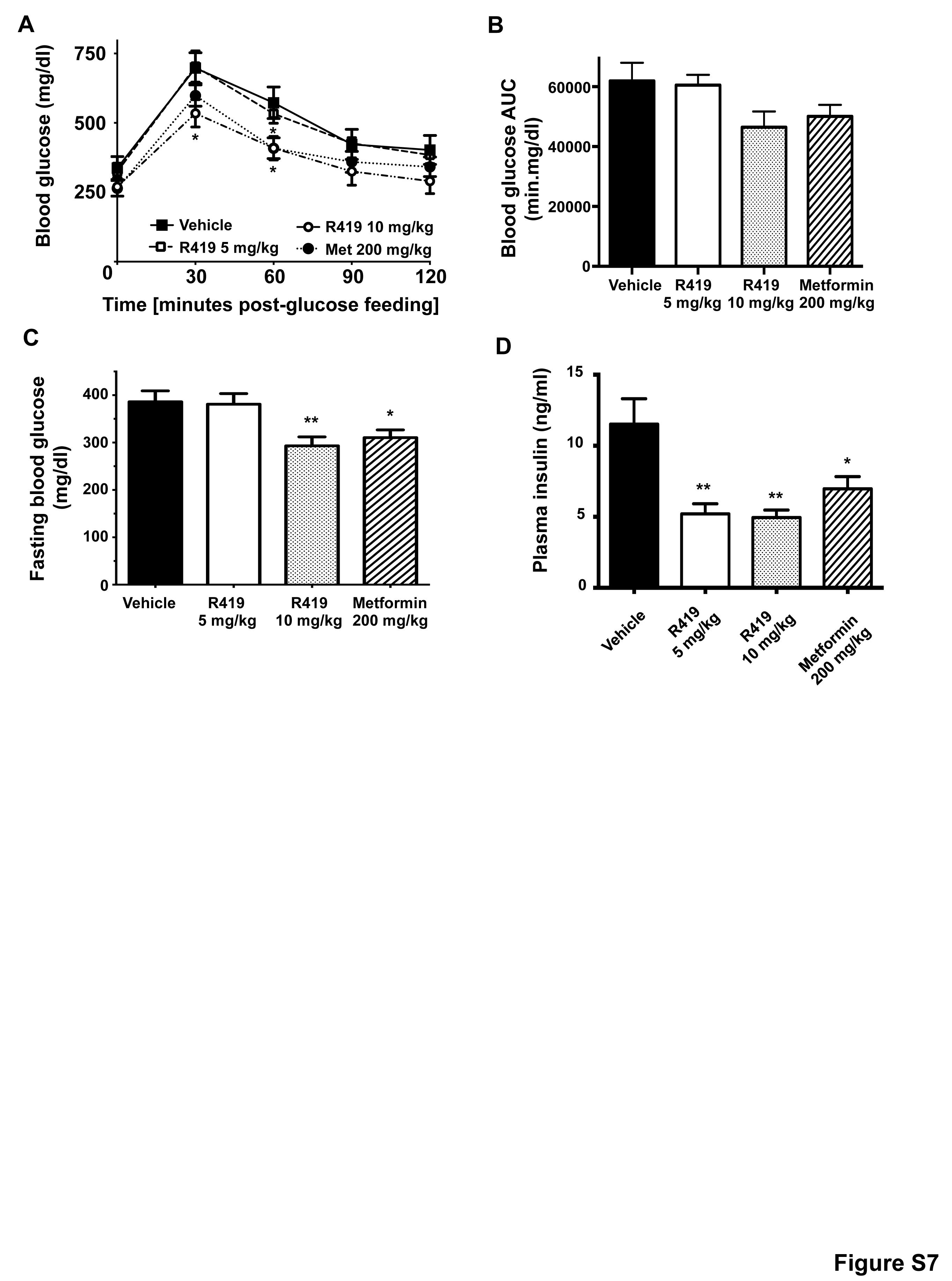

Supplement: Figure S7 — R419 improves glucose tolerance in db/db mice. Male db/db mice (8 weeks old) were PO QD dosed with vehicle, 5 mg/kg, or 10 mg/kg R419, or 200 mg/kg of metformin for 11 days. A: Oral glucose tolerance test (OGTT) following 11-day treatment (n=12/group). OGTT was performed 24 hours after the last dosing. Glucose was administered orally at 2 g/kg following a 6-hour fast with food removal at 5 AM. Repeated measures two-way ANOVA followed by the Dunnett ad-hoc test were performed and the multiple comparison test was done against the corresponding vehicle control at each timepoint. Significant variation among the treatment groups was not reached by repeated measures two-way ANOVA, however, Dunnett’s multiple comparison test showed significance between the R419 10 mg/kg group and the vehicle group at 30 and 60 minute timepoints and between the R419 5 mg/kg group and the vehicle group at 30 minute timepoint. B: Area under the curve corresponding to the graphs shown in A was calculated using GraphPad Prism version 6.0. Statistical significance between R419 and metformin treatment groups and the vehicle control was not reached (Ordinary one-way ANOVA with the Dunnett ad-hoc test, the multiple comparisons against the vehicle control). C: Fasting blood glucose levels following 11-day treatment (n=24/group). Blood sampling was performed 24 hours after the last dosing. Animals were fasted 6-hours prior to the blood sampling. Ordinary one-way ANOVA with the Dunnett ad-hoc test was performed for statistical analysis and the multiple comparison test was done against the vehicle control. D: Plasma insulin following an 11-day R419 treatment (n=12/group). Blood was collected 24 hours after the last compound dose, following a 6-hour fast. The data are presented as mean (symbol or bar) ± SEM (line). Ordinary one-way ANOVA with the Dunnett ad-hoc test was performed for statistical analyses and the multiple comparison test was done against the vehicle control. For all figures, asterisks *, [file pone.0081870.s007.tif]

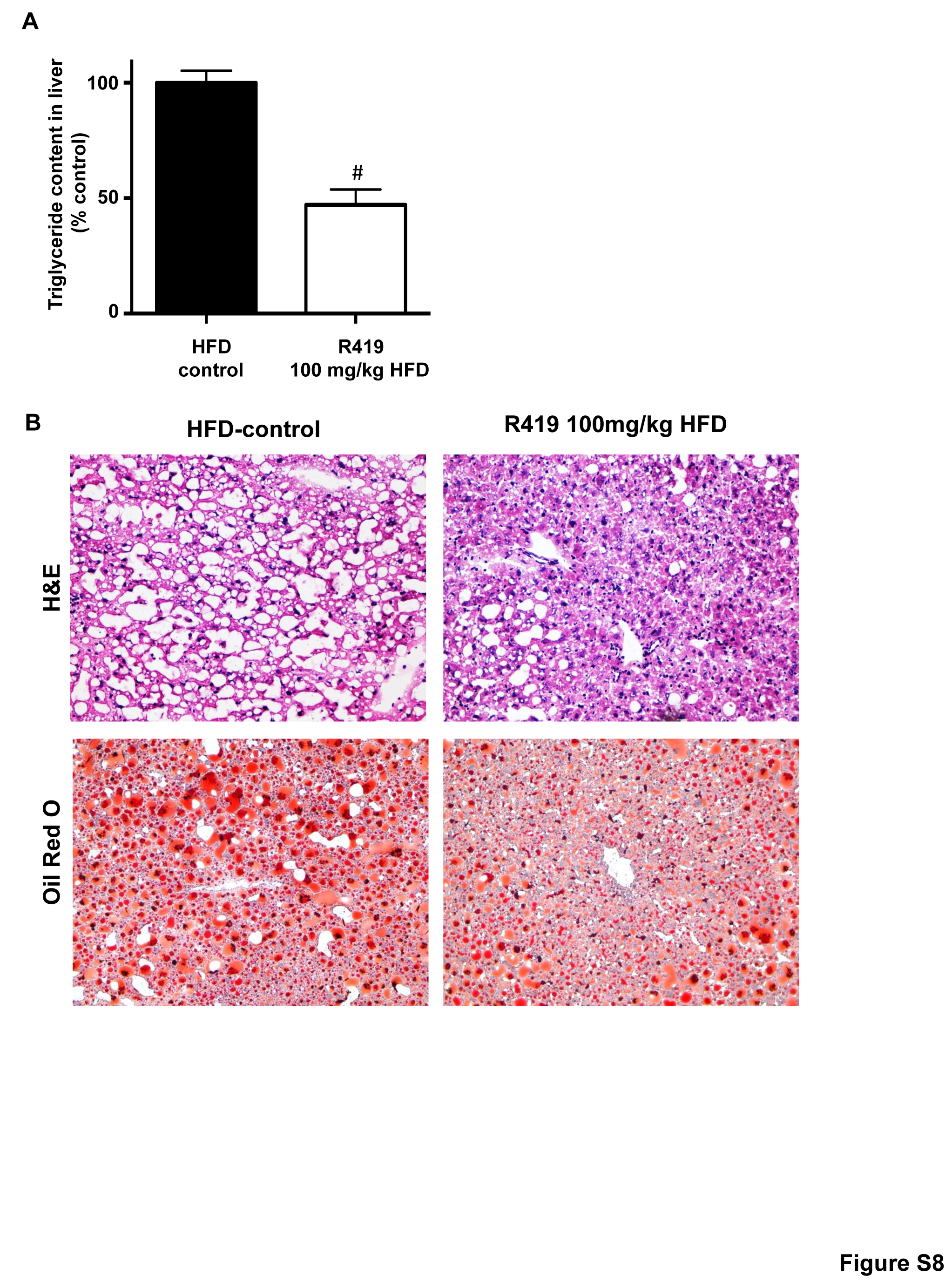

Supplement: Figure S8 — Reduction of steatosis in aged diet-induced obese mice after treatment of R419. Male C57BL6 mice were maintained on a high fat diet (HFD) since 6 weeks of age (diet-induced obesity, DIO). Treatments were started at the 39th week on HFD. R419 was prepared in HFD (D12492, Research Diet, Inc.). During the study, body weights and food intake were comparable between the HFD control and the R419 group. After 10 weeks of compound treatment, livers were harvested for triglyceride content and histological analysis A: Reduction of triglyceride content in liver from R419-treated aged DIO mice. The data are normalized to triglyceride content in aged DIO mice fed with control HFD. Unpaired two-tailed t-tests were performed between HFD control (n=8) and R419-treated group (n=8). Asterisk # represents p < 0.0001. B: Reduced hepatic steatosis in R419 treated mice. Frozen liver sections were stained with hematoxylin and eosin or oil red o dye, and examined by microscopy. Representative images for HFD control and R419 treated mice are shown at a 20 X magnification. (TIF) [file pone.0081870.s008.tif]

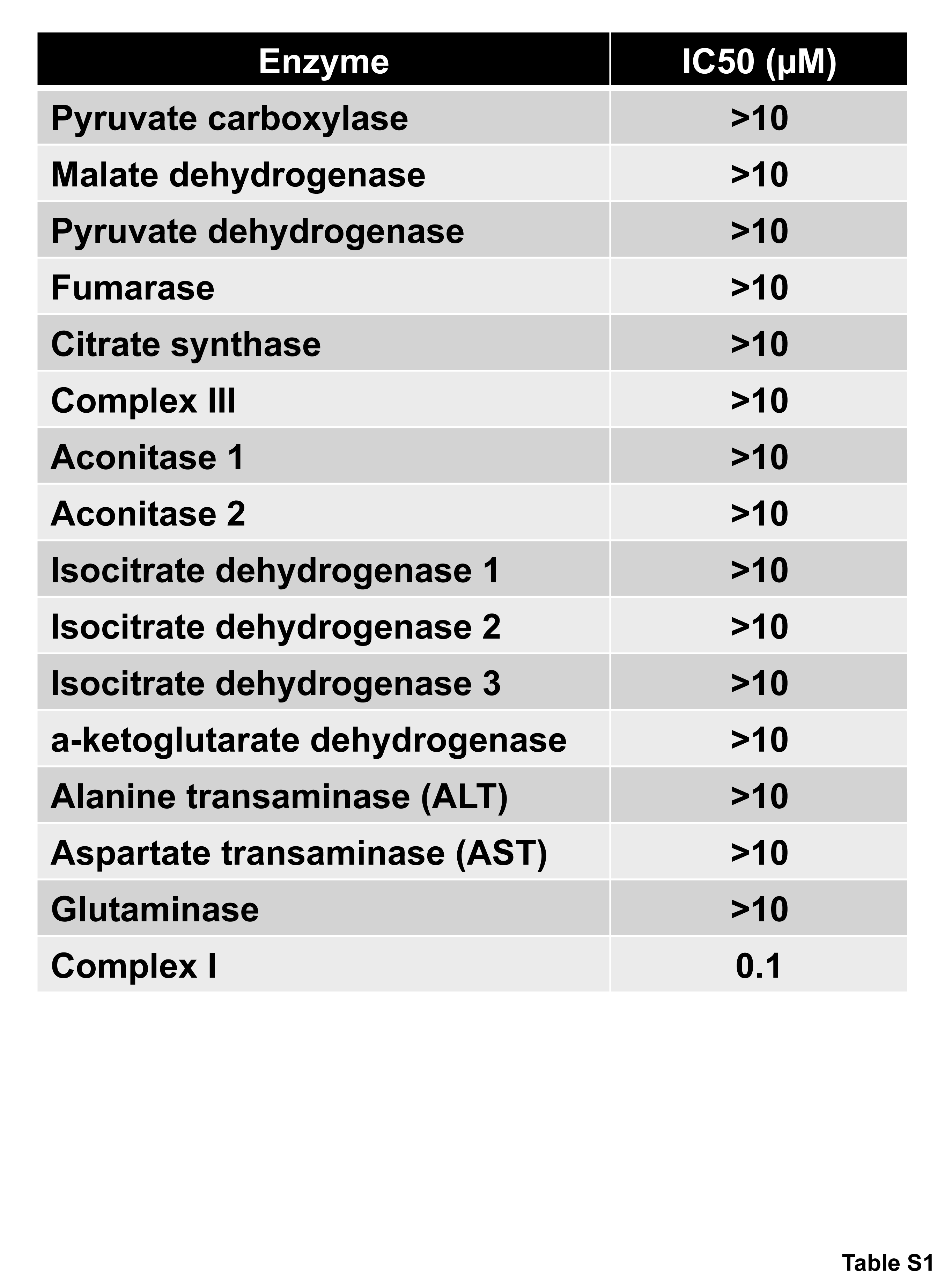

Supplement: Table S1 — No clear inhibitory effects by R419 on activity of TCA enzymes invitro. All the enzymes with the exception of complexes I, III, aconitase 2 and isocitrate dehydrogenases 2 and 3 were purchased from Sigma and tested according to protocols provided by the manufacturer. The rest of the enzyme activities were tested using mitochondrial lysate obtained by extracting purified mitochondria with two volumes of mammalian protein extraction reagent (Pierce) for 1 hour followed by a 10 min high speed spin in a table-top centrifuge. The assay conditions for complexes I, aconitase 2 and isocitrate dehydrogenases 2 and 3 were described elsewhere (S1-S5). Kinetics of NADH/NAD+ or NADPH/NADP+ transition was monitored spectrophotometrically at 340 nm. IC50s were presented as mean of multiple experiments (n≥2). (TIF) [file pone.0081870.s009.tif]
