# Supplementary material for: AMPK Activation through Mitochondrial Regulation Results in Increased Substrate Oxidation and Improved Metabolic Parameters in Models of Diabetes
Source: PLoS One. 2013 Dec 5;8(12):e81870. doi: 10.1371/journal.pone.0081870 (PMC3855387; doi:10.1371/journal.pone.0081870)
Supplement: References S1 — All references cited in SUPPLEMENTAL MATERIALS are listed in a separate supporting information document. (DOCX) [file pone.0081870.s010.docx]

**Supplemental references S1-S5**

S1. Owen M, Doran E, Halestrap A (2000) - Evidence that metformin exerts its anti-diabetic effects through inhibition of complex 1 of the mitochondrial respiratory chain. Biochem J 3: 607-614.

S2. Ward PS, Patel J, Wise DR, Abdel-Wahab O, Bennett BD, et al. (2010) The common feature of leukemia-associated IDH1 and IDH2 mutations is a neomorphic enzyme activity converting alpha-ketoglutarate to 2-hydroxyglutarate. Cancer Cell 17: 225-234.

S3. Gardner P, Nguyen D, White C (1994) - Aconitase is a sensitive and critical target of oxygen poisoning in cultured mammalian cells and in rat lungs. Proc Natl Acad Sci U S A 91: 12248-12252.

S4. Scislowski P, Zolnierowicz S, Zelewski L (1983) - Subcellular distribution of isocitrate dehydrogenase in early and term human placenta. Biochem J 214: 339-343.

S5. Hartong DT, Dange M, McGee TL, Berson EL, Dryja TP, et al. (2008) Insights from retinitis pigmentosa into the roles of isocitrate dehydrogenases in the Krebs cycle. Nat Genet 40: 1230-1234.
